# *Neurospora crassa* Female Development Requires the PACC and Other Signal Transduction Pathways, Transcription Factors, Chromatin Remodeling, Cell-To-Cell Fusion, and Autophagy

**DOI:** 10.1371/journal.pone.0110603

**Published:** 2014-10-21

**Authors:** Jennifer L. Chinnici, Ci Fu, Lauren M. Caccamise, Jason W. Arnold, Stephen J. Free

**Affiliations:** Department of Biological Sciences, SUNY University at Buffalo, Buffalo, New York, United States of America; Georg-August-University of Göttingen Institute of Microbiology & Genetics, Germany

## Abstract

Using a screening protocol we have identified 68 genes that are required for female development in the filamentous fungus *Neurospora crassa*. We find that we can divide these genes into five general groups: 1) Genes encoding components of the PACC signal transduction pathway, 2) Other signal transduction pathway genes, including genes from the three *N. crassa* MAP kinase pathways, 3) Transcriptional factor genes, 4) Autophagy genes, and 5) Other miscellaneous genes. Complementation and RIP studies verified that these genes are needed for the formation of the female mating structure, the protoperithecium, and for the maturation of a fertilized protoperithecium into a perithecium. Perithecia grafting experiments demonstrate that the autophagy genes and the cell-to-cell fusion genes (the MAK-1 and MAK-2 pathway genes) are needed for the mobilization and movement of nutrients from an established vegetative hyphal network into the developing protoperithecium. Deletion mutants for the PACC pathway genes *palA*, *palB*, *palC*, *palF*, *palH*, and *pacC* were found to be defective in two aspects of female development. First, they were unable to initiate female development on synthetic crossing medium. However, they could form protoperithecia when grown on cellophane, on corn meal agar, or in response to the presence of nearby perithecia. Second, fertilized perithecia from PACC pathway mutants were unable to produce asci and complete female development. Protein localization experiments with a GFP-tagged PALA construct showed that PALA was localized in a peripheral punctate pattern, consistent with a signaling center associated with the ESCRT complex. The *N. crassa* PACC signal transduction pathway appears to be similar to the PacC/Rim101 pathway previously characterized in *Aspergillus nidulans* and *Saccharomyces cerevisiae*. In *N. crassa* the pathway plays a key role in regulating female development.

## Introduction

From the viewpoint of a developmental biologist, the study of perithecial development in *Neurospora crassa*, and in the related ascomycetes *Sordaria macrospora*, *Podospora anserina*, and *Gibberella zeae* (anamorph *Fusarium graminearum*) has several advantages [1]. The morphological stages of perithecial development have been carefully cataloged by Lord and Read [2]. The perithecia is a reasonably simple structure with a limited number of different cell types [3]. Large numbers of perithecia can be generated and studied as they go through six days of development in a somewhat synchronous manner [4], [5]. The genomes of these ascomycetes have been sequenced [6], [7]. The expression pattern of the entire *N. crassa* genome has been examined by RNAseq during perithecia maturation [4]. Similarly, genome-wide expression patterns have been extensively analyzed during the development of *S. macrospora* and *Gibberella zeae* [5], [8], [9]. The organisms are haploid except during mating when a dikaryotic ascogenous tissue is generated and a diploid cell, which immediately undergoes meiosis, is formed. The haploid nature of the organisms facilitates the isolation and characterization of mutants affected in female development [1], [10]–[12]. Most importantly, the creation of the *N. crassa* single gene deletion library provides a unique opportunity to carry out a comprehensive genetic analysis of female development in a filamentous fungus [6], [13], [14].

The morphological events that occur during female development have been well documented in *N. crassa*, *S. macrospora*, *P. anserina*, and *G. zeae* [1], [2], [11], [15]–[21]. Female development can be initiated in *N. crassa* by nitrogen deprivation [22]. At the onset of female development a specialized coiled hyphal structure, called an ascogonium, is generated from a vegetative hypha. The ascogonium grows into a tightly woven spherically-shaped structure called a protoperithecium. The protoperithecium contains two types of cells, the ascogenous hyphae in the middle of the structure, and an outer layer of protective hyphae called the peridium. The ascogenous hyphae are generated from the ascogonium, but some of the hyphal elements of the peridium are thought to be generated by hyphae from the vegetative tissue that join with the ascogonium hyphae to create the protective outer layer. A long hypha, termed a trichogyne, is generated from the ascogenous hyphae and grows out of the protoperithecium. The trichogyne is attracted to a pheromone released by conidia and hyphae of the opposite mating type, and is able to undergo a cell fusion event with a cell of the opposite mating type to generate a dikaryon (a cell with two different types of nuclei) [23]. The pheromones and pheromone receptors that function in the chemotrophic growth of the trichogyne to a cell of the opposite mating type have been identified and characterized [24]–[27]. The movement of the male nucleus from the trichogyne into the ascogenous tissue, and the nuclear events within the ascogenous hyphae have been carefully characterized in *N. crassa* by Raju [23]. The male nucleus travels down the trichogyne into the ascogenous hyphae, where it undergoes several rounds of nuclear division. Hyphae having the shape of a “shepherd’s crook” (an upside down J) called crosiers, are generated within the ascogenous tissue. A single ascus cell containing a female and a male nucleus is generated at the top of the “crook” within each of these crosiers. Nuclear fusion occurs in the ascus cell, followed immediately by a meiotic division and a single mitotic division to generate a linear array of four pairs of nuclei. These then mature into eight ascospores. While the male nuclei are being replicated and ascospore formation occurs, the other cell types in the perithecium undergo development. The fertilized perithecium dramatically increases in size and becomes flask shaped. The outer layers of the peridium become highly melanized [10]. Specialized cells called paraphyses, which are thought to help support the development of the ascospores, are generated from the inner layer of the peridium and grow between the developing asci [2]. As perithecium development nears completion, an ostiolar pore (opening) is generated at the top of the flask-shaped perithecium. At the end of female development the mature ascospores are ejected through the ostiolar pore.

During the 1970 s, studies demonstrated that *N. crassa* female development was amenable to genetic analysis. Johnson [10] isolated a large number of mutants affected in female development and ordered these mutants based on the size of the developing protoperithecia. Vigfusson and Weiljer [28], Tan and Ho [29], and Mylyk and Thelkeld [30] also isolated female sterile mutants. Some of the mutations were mapped onto the *N. crassa* genetic map, while others were unmapped. Although these female developmental mutants were deposited in the Fungal Genetics Stock Center, only recently have any of the genes defined by these mutants been identified. Four of these “classical” female developmental genes, *ff-1* (female fertile-1), *fs-n* (female sterile-n), *ty-1/ste-50* (tyrosinaseless-1 or sterile-50), and *per-1* (perithecial-1) were recently identified by whole genome sequencing of the mutant strains [31]. The *ff-1* gene (NCU01543) encodes a LIM domain-containing protein with homology to Pat1p, a topoisomerase II-associated protein. The mutation in the *fs-n* strain was shown to be in the *so/ham-1* gene (NCU02794), a WW domain-containing protein that has been shown to be needed for cell-to-cell fusion as well as for female development [32]. The *per-1* mutation affects the melanization of the perithecia, and the mutant was initially identified by the presence of an unmelanized perithecia [33]. The *per-1* gene (NCU03584) encodes a polyketide synthase, and probably functions in a melanin biosynthetic pathway. The *ty-1/ste-50* mutant has a complex phenotype. It produces short aerial hyphae (flat conidiation), is female infertile, and is “tyrosinaseless” [34]. The *ty-1/ste-50* gene (NCU00455) encodes a homolog of the *S. cerevisiae* Ste50 protein, a scaffold protein for the MAP kinase pathways.

In addition to a large number of *N. crassa* genes that have been identified as being needed for female development, many additional female development genes have been identified and characterized in *S. macrospora*, *P. anserina*, *Magnaporthe grisea*, and *Aspergillus nidulans* (see review by Pöggeler et al. [1]). These additional genes include transcription factors, signal transduction pathway proteins, and autophagy proteins, and provide a wealth of information on female development in filamentous fungi.

In this report, a morphological screening of the *N. crassa* single gene deletion library was used to identify the genes that are needed for the development of protoperithecia and their subsequent maturation into perithecia. In characterizing the mutants identified in this screening, we identified 68 genes that are needed for female development. One of the more interesting findings from the screening experiments was that the PACC signal transduction pathway is required for female development in *N. crassa*.

## Results and Discussion

### Isolation of protoperithecia-defective mutants and co-segregation experiments

**Figure 1:**
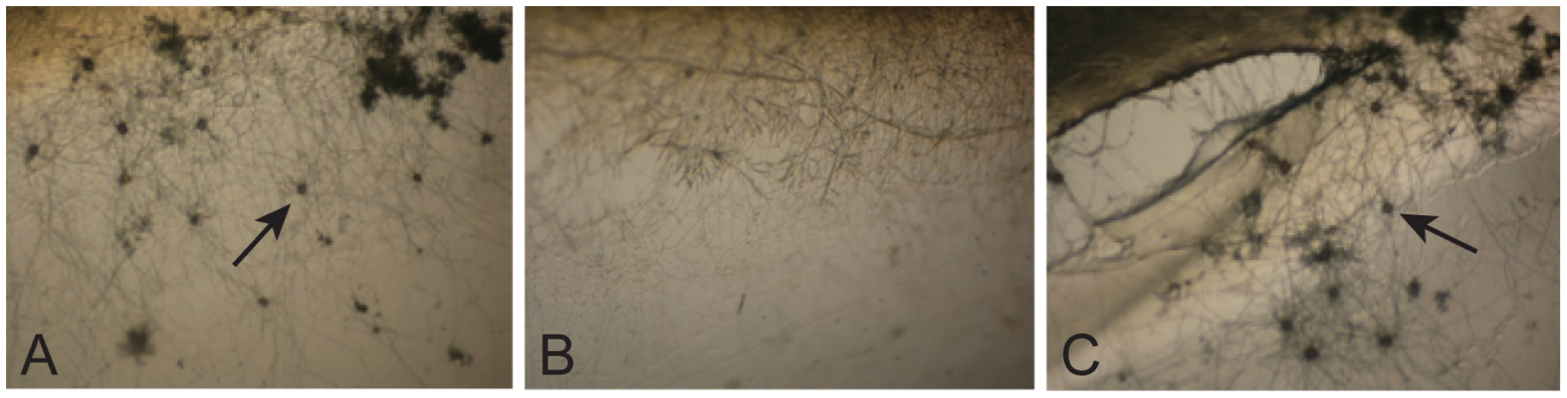
Screening and complementation analysis. Wild type, a Δ*palF* isolate, and a Δ*palF* isolate that has been transformed with a wild type copy of the *palF* gene were grown on 3 ml slants of SCM agar medium for 10 days to allow them to form protoperithecia. The three panels show images of the hyphae and protoperithecia on the test tube glass adjacent to the agar slant. An abundance of protoperithecia are produced by the wild type isolate on the glass at the edge of the agar (first panel/A) while the Δ*palF* mutant is unable to produce protoperithecia (second panel/B). Transformation of the Δ*palF* mutant with a wild type copy of the *palF* gene restores the ability to generate protoperithecia (third panel/C). Arrows point to examples of protoperithecia.

To identify genes that are required for female development in *N. crassa*, a large-scale mutant screening experiment was carried out. As described in Materials and Methods, each of the 10,000 isolates in plates 1 to 119 of the *N. crassa* single gene deletion library was tested for the ability to produce protoperithecia and perithecia on a 3 ml slant of synthetic crossing medium (Figure 1). This screening was done in addition to our previous screening of the library to identify cell-to-cell fusion mutants, which were shown to be defective in protoperithecium development [35]. The purpose of the screening experiments described in this report was to identify other types of genes that are needed for female development. In these screening experiments we identified 649 mutants (representing 508 genes) that were either protoperithecia-defective or perithecia-defective. Co-segregation experiments were done for 443 of these genes. Co-segregation experiments were not done for most of the genes that encoded a known function not directly related to female development (genes important for mitochondrial ATP synthesis, ribosome functions, vesicular trafficking, etc.; see Table S1) and genes where the library contained two deletion isolates and only one of the isolates displayed the mutant phenotype. These co-segregation experiments showed that for 123 of the genes, the mutant phenotype co-segregated with the gene deletion, suggesting that the deletion might be responsible for the female-defective phenotype. Included in these genes were 31 genes that had been previously identified as being required for *N. crassa* female development (see Tables 1–3). This demonstrates that the screening and co-segregation experiments effectively identified genes that function in the process of female development. For 320 of the genes identified in the screening experiments, the co-segregation experiments showed that the female developmental defect did not co-segregate with the gene deletion, indicating that these mutants had additional mutations that were responsible for the mutant phenotype. Excel files showing the results of our screening, co-segregation, complementation, and RIP experiments can be accessed at our website (http://www.biology.buffalo.edu/Faculty/Free/KO_list_2014/KO_List.html).

**Table 1:** Genes from the PacC pathway are required for female development.

Genes	NCU#	Co-segregation	Complementation	Reference information
*pacC*	00090	Yes	Yes	This report
*palA*	05876	Yes	Yes	This report
*palB*	00317	Yes	Yes	This report
*palC*	03316	Yes	Yes	This report
*palH*	00007	Yes	Yes	This report
*palF*	03021	Yes	Yes	This report

A notation of “This report” in the reference information column indicates that the gene was identified as being needed for *N. crassa* development by our experiments.

**Table 2:** Signal transduction pathway genes are required for female development.

Gene name	NCU #	Co-segregation	Complementation	Reference information
		MAP kinases		
*mik-1*	02234*	Yes	PP	Maerz et al. [110]; Park et al. [105]
*mek-1*	06419*	Yes	PP	Maerz et al. [110]; Park et al. [105]
*mak-1*	09842*	Yes	PP	Maerz et al. [110]; Park et al. [105]
*nrc-1*	06182*	Yes	PP	Kothe and Free [64]; Maerz et al. [110];Pandey et al. [63]
*mek-2*	04612*	Yes	PP	Maerz et al. [110]; Pandey et al. [63]
*mak-2*	02393*	Yes	PP	Maerz et al. [110]; Pandey et al. [63]; Li et al. [111]
*os-2*	07024	Yes	PP	Lichius et al. [45]
*os-4*	03071	Yes	PP	Lichius et al. [45]
*os-5*	00587	Yes	PP	Lichius et al. [45]
		Genes encoding MAPKpathway components		
*ste-50 or ty-1*	00455	Yes	PP	McCluskey et al. [31]
*hym-1*	03576	Yes	PP	Dettmann et al. [112]
*rac-1*	02160*	Yes	PP	Araujo-Palomares et al. [113];Fu et al. [35]
*rho-1*	01484	NA	PP	Richthammer et al. [114]
*rgf-1*	00668	NA(het)	PP	Richthammer et al. [114]
*lrg-1*	02689	Yes	PP	Vogt and Seiler [115]
*pp-2A*	06563*	Yes	PP	Fu et al. [35]
PP2A activator	03269*	Yes	–	This report
*pp-1*	00340*	NA(het)	PP	Leeder et al. [91]; Li et al. [111]
*so, fs-n* or *ham-1*	02794*	Yes	PP	Fleissner et al. [32]; Engh et al. [116]
*ham-2*	03727*	Yes	PP	Xiang et al. [42]; Bloemendal et al. [75]
*ham-3*	08741*	Yes	PP	Dettmann et al. [43]; Simonin et al. [117]; Bloemendal et al. [118]
*ham-4*	00528*	Yes	PP	Dettmann et al. [43]; Simonin et al. [117]
*mob-3*	07674*	Yes	PP	Dettmann et al. [43]; Maerz et al. [41]
*ham-5*	01789*	Yes	PP	Aldabbous et al. [86]
*ham-6*	02767*	Yes	PP	Fu et al. [35]; Nowrousian [119]
*ham-7*	00881*	Yes	PP	Fu et al. [35]; Maddi et al. [120]
*ham-8*	02811*	Yes	PP	Fu et al. [35]
*ham-9*	07389*	Yes	PP	Fu et al. [35]
*amph-1*	01069*	Yes	PP	Fu et al. [35]
*whi-2*	10518*	Yes	Yes	This report
*prs-1*	08380*	Yes	Yes	This report
*rrg-1*	01895	–	PP	Jones et al. [121]
		Nox pathway genes		
*nox-1*	02110*	Yes	PP	Cano-Dominguez et al. [69]
*nor-1*	07850*	NA(het)	PP	Cano-Dominguez et al. [69]
		Pheromone signaling genes		
*mfa-1*	16992	NA	PP	Kim and Borkovich [24]
*pre-1*	00138	–	PP	Kim and Borkovich [24]; Poggeler et al. [122]
*ccg-4*	02500	–	PP	Kim and Borkovich [24]
*pre-2*	05758	–	PP	Kim and Borkovich [24]; Poggeler et al. [122]
		Septation initiation network		
*cdc-7*	01335	Yes	PP	Park et al. [48]; Heilig et al. [76]
*sid-1*	04096	–	PP	Heilig et al. [76]
*dbf-2*	09071	–	PP	Heilig et al. [76]
		Calcium signaling		
*cnb-1*	03833	NA	PP	Kothe and Free [64]
*camk-1*	09123	–	–	Park et al. [48]
*ham-10*	02833*	Yes	PP	Fu et al. [35]
		Other signaling pathways		
*gna-1*	06493	NA	PP	Ivey et al. [123]; Kamerewerd et al. [124]
*gnb-1*	00440	Yes	PP	Yang et al. [125]; Kamerewerd et al. [124]
*gng-1*	00041	NA	PP	Krystofova et al. [126]; Kamerewerd et al. [124]
*cpc-2*	05810	NA(het)	PP	Müller et al. [127]
*fi*	04990	Yes	PP	McCluskey et al. [31]
*stk-22*	03523	Yes	Yes	Park et al. [48]; This report
*stk-16*	00914	Yes	–	Park et al. [48]
*div-4*	04426	Yes	Yes	Park et al. [48]; This report
*stk-47*	06685	Yes	–	Park et al. [48]

PP – Previously published data demonstrated that the gene was needed for female development.

NA – a deletion strain is not available in the single gene deletion library.

NA(het) – the deletion strain in the single gene deletion library is a heterokaryon and a homokaryon isolate was not available during the screening experiments.

RIP – a RIP experiment was used to verify that the gene is required for female development.

An * by the NCU number indicates that the gene is needed for CAT (conidia anastomosis tube) formation (a cell fusion phenotype) and is likely to be a component of either the MAK-1 or MAK-2 signal pathway.

A notation of “This report” in the reference information column indicates that the gene was either newly identified or verified by co-segregation and complementation analysis as being needed for *N. crassa* development by our experiments.

**Table 3:** Transcription factors needed for female development.

Gene	NCU#	Co-segregation	Complementation	Reference information
*pacC*	00090	Yes	Yes	This report
*asm-1*	01414	NA (het)	PP	Aramayo et al. [85]
*rco-1*	06205	Yes	PP	Yamashiro et al. [128]; Aldabbous et al. [86]
*rcm-1*	06842	NA (het)	PP	Kim and Lee [129]; Aldabbous et al. [86]
*ada-1*	00499	Yes	Yes	Colot et al. [84]; This report
*adv-1*	07392	Yes	Yes	Colot et al. [84]; Fu et al. [35]
*fsd-1*	09915	Yes	PP	Hutchinson and Glass [90]
*fmf-1*	09387	–	PP	Iyer et al. [89]
*mcm-1*	07430	NA	PP	Nolting and Poeggeler [93], [94]
*pp-1*	00340	NA (het)	PP	Leeder et al. [91]

PP – Previously published data demonstrated that the gene was needed for female development.

NA – a deletion strain is not available in the single gene deletion library.

NA (het) – the deletion strain in the single gene deletion library is a heterokaryon and a homokaryon isolate was not available during the screening experiments.

Among the mutants we identified in the screening experiment were several mutants that grew very poorly and had mutations in mitochondrial proteins, in general transcription proteins, in protein translation functions, in vesicular trafficking, and other “general cellular health” functions. Co-segregation experiments on a few of these mutants showed that the “general health” mutation did co-segregate with the female-defective phenotype. A list of these genes is found in Table S1, and, for the most part, these genes were not further characterized in our study. These mutants demonstrate that the hyphae must be “healthy” in order to participate in female development. We also noted that our experiments identified 18 genes that function in chromatin assembly and remodeling. The co-segregation experiments, along with some complementation experiments, clearly demonstrated that chromatin assembly and remodeling are required for female development. Others have previously shown chromatin assembly and remodeling mutants are defective in female development [36]–[39]. We have listed these chromatin organization genes in Table S2. Although a study of how chromatin remodeling is involved in directing female development is a very interesting topic, we did not focus on these mutants in our current study.

### Complementation and RIP experiments

Complementation or RIP experiments were carried out on a majority of the putative female developmental genes defined by the co-segregation experiments. However, for some of the genes, pre-existing definitive information showing that the gene was required for *N. crassa* female development had been previously published, and complementation experiments were not carried out on these mutants. In these situations, the publication citation showing that the gene was needed for the protoperithecium development or maturation is provided in Tables 2 through 5. As described in Materials and Methods, the complementation experiments used wild type copies of the putative female developmental genes to transform the deletion mutants. The ability of the wild type gene to restore a wild type phenotype to a deletion mutant was taken as definite proof that the gene was needed for female development.

**Table 4:** Autophagy genes required for female development.

Gene	NCU#	Co-segregation	Complementation	Reference information
*atg-3*	01955	Yes	Yes	This report
*atg-8*	01545	Yes	Yes (RIP)	Fu et al. [35]; Voigt et al. [97]; Liu et al. [100]
*atg-12*	10049	Yes	–	This report
*atg-7*	06672	Yes	–	Nolting et al. [98]; This report
*atg-9*	02422	Yes	–	This report
*atg-10*	02779	Yes	–	This report
*atg-1*	00188	Yes	–	This report
*atg-5*	04662	Yes	–	This report
*atg-13*	04840	Yes	–	Kikuma and Kitamoto [101]; This report
*atg-18*	03441	Yes	–	This report

RIP – a RIP experiment was used to verify that the gene is required for female development.

A notation of “This report” in the reference information column indicates that the gene was identified as being needed for *N. crassa* development by our experiments.

**Table 5:** Miscellaneous genes needed for female development.

Gene	NCU#	Co-segregation	Complementation	Reference information
Tyrosinase	00776	NA	PP	Fuentes et al. [102]
*per-1* polyketide synthase	03584	NA(het)	PP	McCluskey et al. [31]
Aldo-keto reductase	01703	Yes	Yes	This report
*fem-1* Hypothetical	03589	Yes	Yes	This report
*fem-2* Hypothetical	07135	Yes	Yes	This report
*fem-3* Hypothetical	03588	Yes	Yes	This report
*fem-4* Hypothetical	06243	Yes	Yes	This report
*fem-5* Hypothetical	02073	Yes	Yes	This report
*fem-6* Hypothetical	09052	Yes	Yes	This report
*fem-7* Hypothetical	03985	Yes	Yes	This report

PP – Previously published data demonstrated that the gene was needed for female development.

NA – a deletion strain is not available in the single gene deletion library.

NA(het) – the deletion strain in the single gene deletion library is a heterokaryon and a homokaryon isolate was not available during the screening experiments.

A notation of “This report” in the reference information column indicates that the gene was identified as being needed for *N. crassa* development by our experiments.

In cases where the complementation experiment was difficult because the mutant did not produce conidia, the cell type used in the transformation experiments, RIP experiments were used to generate additional mutated copies of the gene. In these cases, one or more of the RIP alleles were PCR amplified and sequenced to verify that the mutant allele(s) had multiple RIP mutations. As a result of our co-segregation, complementation, and RIP experiments, we identified 68 genes that are needed for female development. While many of these genes had been previously reported as being needed for female development, our studies identified 32 genes that had not been previously identified as being required for female development in *N. crassa* (denoted as being defined by this report in Tables 1 through 5). The results of our screening, cosegregation, and complementation experiments demonstrate the value of a careful screening of the *N. crassa* single gene deletion library in characterizing the biology of the model filamentous fungus. Our research also highlights the importance of using co-segregation and complementation experiments in verifying that the deletions identified in screening the deletion library give rise to the observed mutant phenotypes. As demonstrated by our study, the presence of secondary mutations in some isolates in the library does not detract from the value and importance of the single gene deletion library in analyzing *N. crassa* gene functions.

### Cell-to-cell fusion assays

To further characterize our mutants, the ability of the mutants to participate in cell-to-cell fusion was assessed with the CAT fusion assay [40]. We and others have previously shown that cell-to-cell fusion is needed for female development [32], [35], [41], [42]. The genes required for cell-to-cell fusion are designated with an asterisk in Table 2. Interestingly, we found that all of the cell-to-cell fusion genes that we identified encode proteins that are either confirmed or likely participants in one of three closely related signaling pathways: 1) the STRIPAK signaling complex, which is required for the movement of MAK-1 into the nucleus in a MAK-2-dependent manner; 2) the MAK-1 cell wall integrity signal transduction pathway; and 3) the MAK-2 hyphal growth signal transduction pathway used to direct the growth of fusion hyphae towards each other [35], [43], [44].

### Ascogonia formation

Female development begins with the formation of the ascogonium, a specialized coiled hyphae structure. To determine whether the mutants were affected in ascogonia formation, mutant conidia were used to inoculate either synthetic crossing medium with sucrose or cellophane filters placed on synthetic crossing medium (without an additional added carbon/energy source), and the formation of ascogonia was assessed under a compound microscope. The PACC pathway mutants (*palA*, *palB*, *palC*, *palF*, *palH*, and *pacC*) had a rather interesting ascogonia formation phenotype. We found that they were unable to produce ascogonia on the synthetic crossing medium with sucrose but were able to form ascogonia on cellophane.

In the ascogonia formation on cellophane experiments, we found that 17 of the mutants were affected in the formation of ascogonia. These included the mutants for the OS MAP kinase pathway gene *os-2*, *os-4*, and *os-5* genes, which have been previously shown to be defective in ascogonia formation by Lichius et al. [45]. We also found that deletion mutants for the transcription factor *rco-1* (NCU06205) and 3 calcium signaling genes, *cnb-1* (NCU03833), *camk-1* (NCU09123), and *ham-10* (NCU02833) were affected in ascogonia formation. This suggests that Ca++ signaling may be required for the initiation of female development in *N. crassa*. Previous work on each of these genes has shown that they are needed for female development [46]–[48]. The other 10 genes affecting ascogonia formation were *rac-1* (NCU02160), *div-4* (NCU04426), *stk-16* (NCU00914), *stk-22* (NCU03523), *stk-47* (NCU006685), *fem-1* (NCU03589), *fem-4* (NCU06243), *fem-5* (NCU02073), *fem-6* (NCU09052), and *fem-7* (NCU03985). Most of these mutants produced an abundance of conidia instead of making ascogonia on the cellophane filter. These mutants also shared a second phenotypic characteristic, a hyper-production of conidia when grown on slants containing synthetic crossing medium with sucrose. We hypothesize that these genes may function in allowing the fungus to choose between two alternative developmental pathways, a sexual pathway leading to female development and the asexual pathway leading to conidiation. These genes may well define a signal transduction pathway needed for initiating female development.

### Perithecium grafting experiments

**Figure 2:**
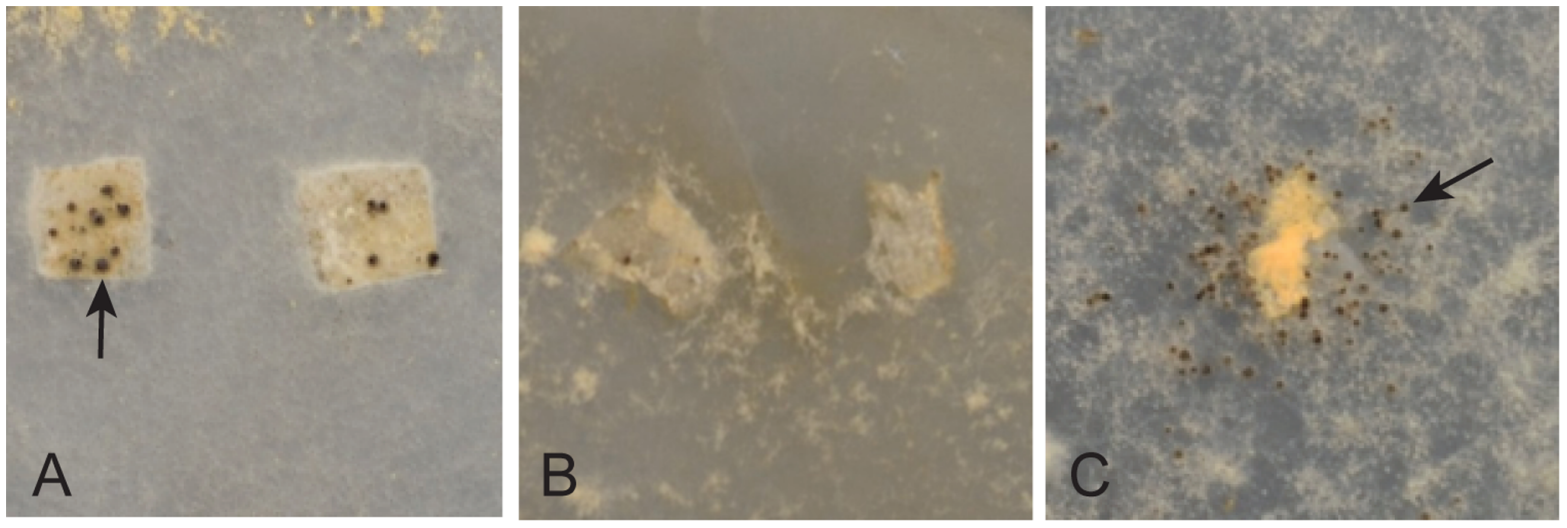
Perithecia grafting experiments. Small pieces of cellophane containing “graft” fertilized wild type protoperithecia were placed on mutant “host” vegetative hyphal networks grown on synthetic crossing medium. The “host” shown are: 1) Δ*fmf-1* (left panel/A) which shows a host supporting the development of the graft perithecia. 2) Δ*ada-1* (middle panel/B) which shows a host not supporting the development of the graft perithecia. 3) Δ*palA* (right panel/C) which shows the graft inducing protoperithecia in the Δ*palA* host. Arrows point to examples of perithecia on the cellophane (left panel/A) and protoperithecia induced in the host vegetative hyphal network (right panel/C).

Female development on synthetic crossing medium occurs in response to nitrogen limitation, a condition in which the fungus may be restricted in the synthesis of new amino acids, and need to rely on pre-existing amino acids for the synthesis of new proteins. We developed a perithecia grafting assay (see Materials and Methods) that allowed us to ask whether the vegetative hyphae from the mutants we identified in our screening experiments were able to support the development of newly fertilized wild type perithecia (Figure 2). The results of the perithecia grafting experiments were quite instructive. We found that deletion mutants for virtually all of the genes known to be needed for autophagy and deletion mutants for all of the genes known to be components of the MAK-1 and MAK-2 signal transduction pathways needed for cell-to-cell fusion were defective in supporting the development of wild type perithecia in these grafting experiments (Figure 2). This demonstrates that both autophagy and cell-to-cell fusion are needed within the vegetative hyphal network. We conclude that the release of amino acids, and perhaps other nutrients, from the vegetative hyphal network and the transferring of these nutrients into the developing perithecium are needed to support female development.

In doing the perithecia grafting experiments, we found that the mating types of the graft and host vegetative network had to be the same in order for the host to be able to support graft development, suggesting that the vegetative incompatibility system is operating between the host and graft tissues. In addition to providing an assessment of whether a mutant “host” supported a wild type “graft”, we noted a second unexpected phenomenon in the grafting experiments. When testing the PACC pathway mutants (*palA*, *palB*, *palC*, *palF*, *palH*, and *pacC*) as hosts, we found that the presence of wild type perithecia grafts of either mating type were able to induce protoperithecia formation in the host hyphal network surrounding the graft (Figure 2). We conclude that the PACC pathway operates to allow the fungus to choose the female developmental pathway in response to environmental/nitrogen limitation cues present in synthetic crossing medium. However, the fungus apparently has other pathways for inducing female development, and one of these pathways is responsive to the presence of nearby perithecia.

### Functional grouping of the genes required for female development

An examination of the genes defined by the deletion mutants showed that we could assign the genes into five different categories. These were: 1) Genes encoding proteins which have been found to function in the PACC signal transduction pathway; 2) Genes encoding proteins that are likely to function in other signal transduction pathways, including the three *N. crassa* MAP kinase pathways, the STRIPAK pathway, the pheromone-responsive pathway, the NOX pathway, the heterotrimeric G protein signaling pathway, the septation initiation network (SIN), a Ca++ signaling pathway, and perhaps other signaling pathways; 3) Genes encoding probable transcription factors; 4) Genes that are required for autophagy; and 5) a few miscellaneous genes that don’t fit into the other categories. These genes are listed in Tables 1 through 5 respectively, along with details about the mutant phenotypes. To provide a somewhat comprehensive gene list, we have included a number of additional genes that have been shown by others to be involved in female development. In most of these cases, the deletion mutants for these genes weren’t identified in our screening experiments because they were not available in the library or they were found as heterokaryons in the library. These “added” genes include *mfa-1*(NCU06992), *pre-1* (NCU00138), *ccg-4* (NCU02500), *pre-2* (NCU05758), *asm-1* (NCU01414), *fmf-1* (NCU09387), *pp-1* (NCU00340), *rho-1* (NCU01484), *nor-1* (NCU07850), *gna-1* (NCU06493), *gng-1* (NCU00041), *cnb-1* (NCU03833), *cpc-2* (NCU05810), *mcm-1* (NCU07430), *per-1* (NCU03584), *rgf-1* (NCU00668), *rrg-1* (NCU01895), *rcm-1* (NCU06842) and *tyrosinase* (NCU00776). Citations for the publications that demonstrated that these genes play roles in female development are included in Tables 2, 3, and 5. We will examine each category of mutants one at a time and describe their phenotypes and how they might function in supporting or directing female development.

### Category #1: The PACC signal transduction pathway

**Figure 3:**
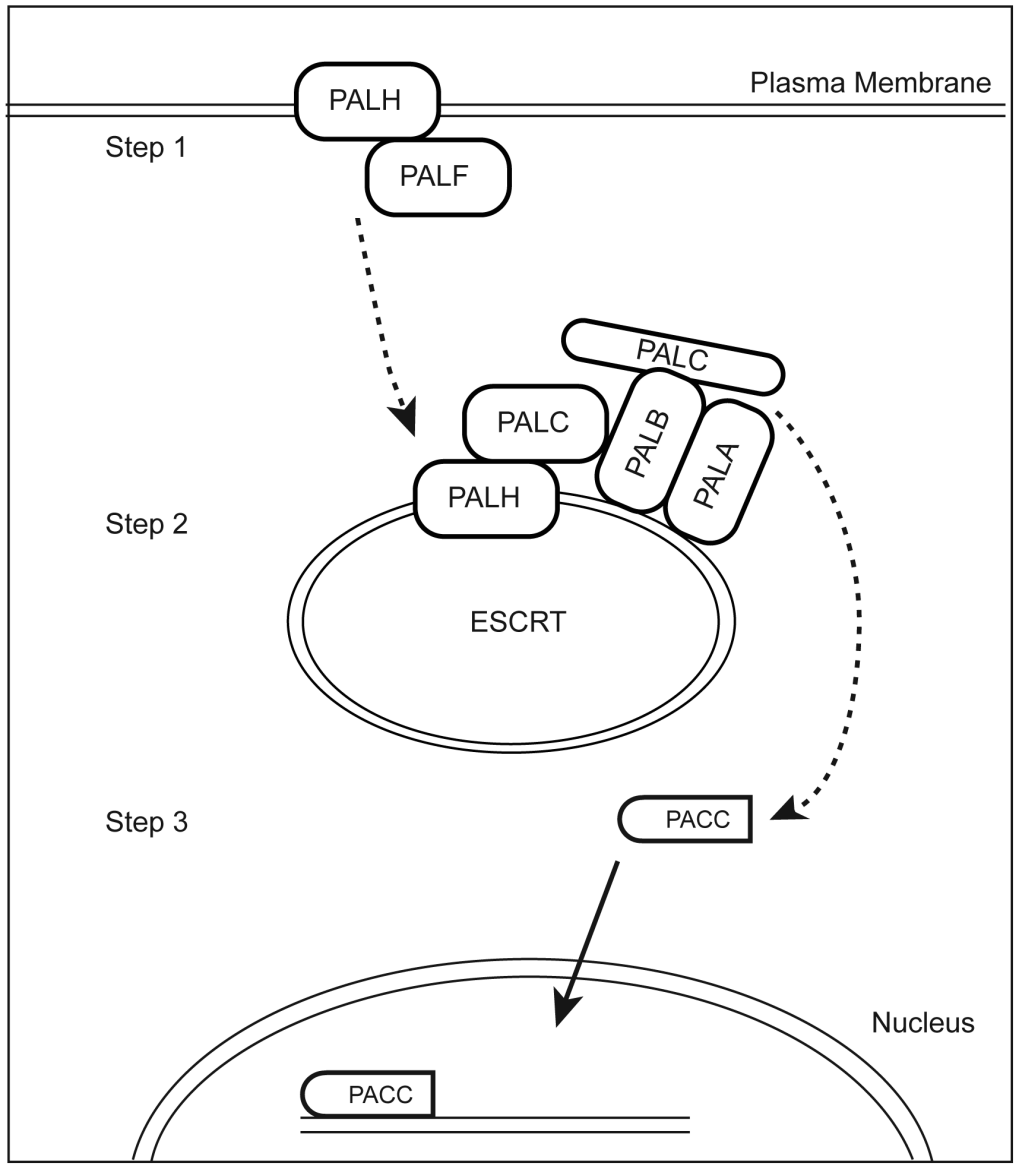
Schematic representation of the *N. crassa* PACC pathway. The PACC signal transduction pathway elements found in *N. crassa*, and the model for how the pathway might function are depicted. The PALH and PALF proteins are thought to be found at the plasma membrane. PALH is a seven transmembrane receptor which is sensitive to environmental cues. PALF is an arrestin type protein that associates with PALH. PALF is phosphorylated and ubiquitinated in response to the environmental cues. These events lead to the endocytosis of the PALH/PALF complex. Following endocytosis, the PALH is directed into an ESCRT compartment, where it enters into a signaling complex containing PALA, PALB, PALC, and PACC. Within the signaling complex, PALB functions as a protease which cleaves PACC. This cleavage event removes a C-terminal inhibitory domain from the PACC transcription factor, and the processed PACC is released from the signaling complex. The activated PACC then enters the nucleus and directs transcriptional activity leading to the formation of the protoperithecium.

The PacC/rim101 signal transduction pathway has been well characterized in *A. nidulans* and *S. cerevisiae*, where the pathway is regulated by the pH of the medium [49]–[53]. A representation of the pathway with the components we have identified in *N. crassa* is shown in Figure 3. In *S. cerevisiae* and *A. nidulans*, the pathway is activated at neutral-to-alkaline pHs, and the PACC protein functions as a transcription factor to activate expression of a number of genes needed for growth under neutral-to-alkaline pH conditions. The PALH protein, a seven transmembrane protein found in the plasma membrane, functions as the receptor or pH sensor for the pathway. PALH is found in a complex with the PALF protein, which has homology to arrestins. Under activating pH conditions, the PALF protein is phosphorylated and ubiquitinated. These modifications to PALF lead to the endocytosis of PALH protein, which joins with the PALA, PALB, PALC, and PACC proteins to create a multimeric signaling center within the ESCRT (endosomal sorting complexes required for transport) complex. Within the signaling center, PALB functions as a processing protease and cleaves an inhibitory C terminal domain from the PACC transcription factor. The activated PACC is released from the ESCRT complex and enters the nucleus. Since the PACC pathway has been characterized as a pH-dependent pathway, finding that it was required for female development in *N. crassa*, which occurs during nitrogen deprivation, was unexpected.

We found that the *ΔpalA*, *ΔpalB*, *ΔpalC*, *ΔpalF*, *ΔpalH*, and *ΔpacC* mutants are all defective in protoperithecium development. We were unable to identify any ascogonia, the initial stage of protoperithecial development, when examining mutant hyphae growing on synthetic crossing medium, while ascogonia and protoperithecia were observed in wild type hyphae. Complementation analysis for these PACC pathway mutants verified that each of the genes was needed for protoperithecium formation (Figure 1, Table 1). The PacC pathway has been well-defined in *A. nidulans*, *S. cerevisiae*, and *C. albicans* as being required to regulate genes involved in growth in neutral-to-alkaline media [49]–[52], [54]–[60]. To the best of our knowledge, this is the first report demonstrating that the pathway is required for protoperithecial development. However, the PacC mutant of *Sclerotinia sclerotium* has been found to be defective in the formation of sclerotia, melanized structures that can remain dormant for many years and give rise to apothecia, the female structure for this species [61].

As noted in the ascogonia formation and perithecia graft experiments, the PacC pathway deletion mutants were unable to generate ascogonia and protoperithecia on synthetic crossing medium, but could produce protoperithecia in response to a signal from nearby fertilized perithecia from either mating type (Figure 2) or when grown of cellophane. We also tested for protoperithecia production on corn meal agar, a medium containing complex carbohydrates which can be used for *N. crassa* matings, and found that the PACC mutants produced protoperithecia on this medium. Our data shows that the PACC pathway is needed for the induction of female development (ascogonial development) only when female development is induced by growth on synthetic crossing medium, a medium that is generally ascribed as inducing female development in response to nitrogen deprivation, but that other environmental cues can induce female development in PACC pathway mutants. It is interesting to note that the environmental conditions inducing female development differ for different fungal species [1], which may reflect differences in their life cycles. We hypothesize that PACC induction of ascogonia production may be restricted to those fungi that induce female development in response to nitrogen limitation.

**Figure 4:**
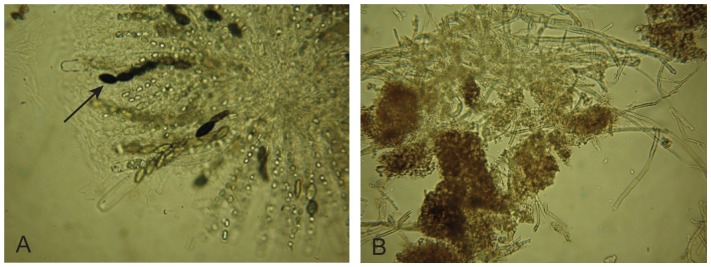
The PACC pathway is required for the development of asci. Fertilized perithecia from wild type (left panel/A) and Δ*palA* (right panel/B) were allowed to develop for 7 days. The perithecia were squashed between a glass slide and a glass coverslip and examined with a transmitted light microscope. The wild type perithecia has generated ascospores (arrow) while the Δ*palA* peirthecia is defective in ascospore formation. The arrow in the left panel points to an ascospore.

To determine whether the protoperithecia produced by PacC pathway deletion mutants were fully functional, we fertilized protoperithecia that had been induced by the presence of nearby perithecia or growth on corn meal agar. Upon fertilization, the mutant perithecia increased in size and began to melanize. However, the perithecia did not complete development and eject ascospores. Several of these mutant perithecia were examined, and we found that the perithecia did not grow as large as wild type perithecia. They also did not become as melanized as wild type perithecia. Microscopic examination of squashed mutant perithecia shows that they do not generate asci (Figure 4). Thus, we find that the PACC pathway is used at two different stages of female development, first during the initial induction of protoperithecial development in response to environmental cues, and then later in the maturing perithecia during the development of asci from the ascogenous tissue.

**Figure 5:**
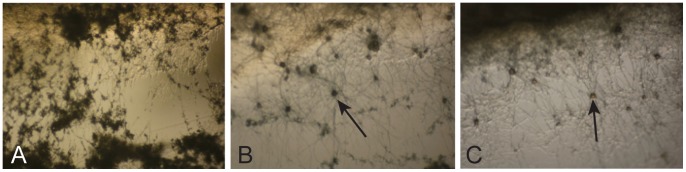
Constitutively active PACC activates female development. Cells were inoculated onto agar slants and allowed to grow for 10 days at room temperature. The left panel (A) shows a Δ*palC* isolate on Vogel’s sucrose medium. The middle panel (B) shows a Δ*palC* isolate that has been transformed with the constitutively activated PACC construct growing on synthetic crossing medium. The right panel (C) shows a Δ*palC* isolate that has been transformed with the constitutively activated PACC construct growing on Vogel’s sucrose medium, a medium that represses female development. Note that the constitutively activated PACC caused protoperithecia production in the absence of PALC on both media. The arrows in the middle and right panels point to protoperithecia.

To determine whether the removal of the C terminal inhibitory domain of PACC was sufficient to direct *N. crassa* cells into the female developmental pathway, we prepared a version of *pacC* in which a stop codon was inserted into the gene at amino acid 492. The truncated protein made from this construct would lack the predicted inhibitory C terminus domain, and would be predicted to give rise to a constitutively active form of PACC. Transformation of wild type isolates with the plasmid encoding this constitutively active PACC resulted in the abundant formation of protoperithecia, even in the Vogel’s medium where female development is normally repressed. Transformants of the Δ*palA*, Δ*palB*, Δ*palC*, Δ*palF*, and Δ*palH* with plasmid encoding the constitutive active form of PACC, also produced an abundance of protoperithecia (Figure 5). This demonstrates that PALA, PALB, PALC, PALF, and PALH are all upstream of PACC within the signaling pathway. We conclude that the activation of PACC is a major event in triggering female development in *N. crassa*, and that activation of PACC is sufficient to direct cells to undergo female development, even in the absence of the normal nitrogen limitation cue.

**Figure 6:**
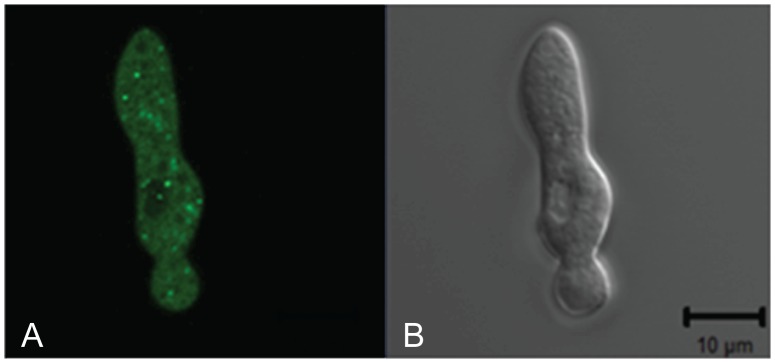
PALA is localized to the small intracellular vesicles. GFP-tagged PALA was expressed under the regulation of the *ccg-1* promoter in a Δ*palA* isolate. The GFP fluorescent image of a germling is shown in the panel on the left (A). The DIC image of the same germling is shown in the right panel (B). The bar in the DIC image is 10 µm in length.

To examine whether the PACC pathway has the same characteristics in *N. crassa* that have been previously identified in the *A. nidulans* and *S. cerevisiae* systems, we decided to examine the intracellular location of PACC pathway components in wild type and mutant isolates. Endogenous promoter-driven GFP-tagged versions of PALA, PALC, and PALH were prepared using the pMF272 vector [62]. The GFP-tagged versions of PALC and PALH did not complement the deletion mutants and we were unable to detect a GFP signal, suggesting that the GFP-tagged proteins were rapidly degraded. However, the GFP-tagged PALA fully complemented the deletion mutation and gave a faint, but detectable intracellular signal in conidia and germlings. The faint signal was localized in a punctate pattern near the periphery of the conidia and germlings. We were unable to detect a signal from the GFP-tagged PALA within early developing protoperithecia (ascogenous coils), suggesting that the signal was weak. We also prepared a *ccg-1* promoter-driven GFP-tagged PALA vector as described in Materials and Methods. Transformation with the *ccg-1* promoter-driven GFP-tagged *palA* provided for a stronger signal in wild type and *ΔpalA* isolates (Figure 6). Examination of the GFP-tagged PALA in germinating conidia showed that the protein was localized in a punctate pattern near the cell surface. The data suggests that the *N. crassa* PACC pathway is localized to the ESCRT complex, just as was previously shown to occur for the *A. nidulans* and *S. cerevisiae* pathways [51], [52]. Our findings are consistent with the *N. crassa* PACC pathway functioning like the canonical PACC pathway defined in *A. nidulans* and *S. cerevisiae*. In *S. cerevesiae*, the PACC homlog, Rim101p, has been shown to be required for meiosis [55], [57], and the inability of the PACC pathway mutants to produce asci would suggest that the *N. crassa* PACC pathway may function in an analogous manner during the later stages of female development.

### Category #2: Genes encoding other signal transduction pathway elements

Table 2 lists 53 genes that are likely components of signal transduction pathways. An extensive analysis of the serine-threonine kinases encoded in the *N. crassa* genome was recently published, and many of the kinases we identify in Table 2 were also found to be defective in female development in that study [48]. Fourteen of these signal transduction pathway genes were previously defined as being required for cell-to-cell fusion in our previous study [35]. All three of the MAP kinase pathways encoded in *N. crassa* genome have been previously shown to be needed for female development [45]. Table 2 contains 25 genes required for the production of CATs (conidial anastomosis tubes), the cell type needed for cell-to-cell fusion. Most of these genes are known to be components of either the MIK-1/MEK-1/MAK-1 cell wall integrity pathway, the NRC-1/MEK-2/MAK-2 hyphal growth pathway, or the NOX pathway. These pathways have been identified as being needed for cell-to-cell fusion [32], [35], [45], [63], [64]. Table 2 contains three newly identified genes, *pp-2A activator* (NCU03269), *whi-2* (NCU10518) and *prs-1* (NCU08380), which are required for the CAT formation and are therefore likely components or regulators of the MAK-1 or MAK-2 pathways. The PP-2A activator (NCU03269) is likely associated with PP-2A (NCU06563), which has been shown to be a member of the STRIPAK complex that regulates the movement of MAK-1 into the nucleus [43]. WHI-2 is a homolog of a yeast general stress regulator [65]–[68]. Recent work in our laboratory has shown that MAK-1 and MAK-2 pathways are affected in the *N. crassa whi-2* mutant [47]. PRS-1 is a putative membrane-associated protein phosphatase and forms a complex with WHI-2 [65]. In yeast, Whi2p and Psr1p have roles in autophagy and in responding to mitochondrial dysfunction [68]. Given the probable association between the PP2A activator and the STRIPAK complex and between PRS-1 and WHI-2, it seems likely that PP2A activator and PRS-1 are also involved in regulating the MAK-1 and MAK-2 pathways in *N. crassa*. Those signal transduction pathway components found in Table 2 that do not affect CAT formation are unlikely to be part of the MAK-1 and MAK-2 pathways, and are more likely to be functioning in some other pathway.

We noted that our list of signal transduction genes included the *nox-1* (NCU02110) and *nor-1* (NCU07850) genes, which function in superoxide production and signaling [69]–[71]. These genes have been previously identified by others as being needed for protoperithecia formation, and may play a key role in cell-to-cell signaling during female development [69], [72], [73].

The *mfa-1* (NCU016992) and *ccg-4* (NCU02500) pheromone genes and the genes encoding their receptors, *pre-1* (NCU000138) and *pre-2* (NCU005758), have been previously identified as playing vital functions during mating and perithecium maturation in *N. crassa* and *S. macrospora* [24], [27], [74]. The PRE-1 and PRE-2 receptors have been previously shown to function in directing growth of the trichogyne toward conidia of the opposite mating type to facilitate the fertilization event [27]. The signal transduction pathway(s) through which the PRE-1 and PRE-2 receptors function has not been characterized. Further analysis will be needed to characterize how this pathway functions.

Mutants that are affected in the process of septation formation have been previously shown to be unable to generate protoperithecia in *S. macrospora* and *N. crassa* [41], [75]–[77]. Our screening experiments corroborate the need for septation during female development. We identified *cdc-7* (NCU01335), *sid-1* (NCU04096), and *dbf-2* (NCU09071) as being needed for protoperithecia formation (Table 2). Several other genes encoding components of the *N. crassa* sepatation initiation network (SIN) have been shown to needed for female development [76]–[80], but the deletion mutants were either absent from the single gene deletion library or found as heterokaryons, and these genes are not listed in Table 2.

Table 2 lists three genes involved in calcium signaling that were identified in our experiments. These are the gene for the calcineurin subunit b, *cnb-1* (NCU03833), the calcium/calmodulin-dependent protein kinase *camk-1* (NCU09123), and *ham-10* (NCU02833), which encodes a C2 domain-containing protein. C2 domains are thought to function as calcium-dependent lipid-binding domains involved in vesicular trafficking. All three of these genes have been previously identified as affecting female development [46]–[48].

In addition to the components of the three MAP kinase pathways, the PACC pathway, the NOX-1 pathway, the septation initiation network, the pheromone pathway, and the calcium signaling pathway, Table 2 also contains five additional kinases. These kinases are *fi* (NCU04990), *div-4* (NCU04426), *stk-16* (NCU00914), *stk-22* (NCU03523), and *stk-47* (NCU06685). Four of these kinases, *div-4*, *stk-16*, *stk-22* and *stk-47* are needed for ascogonia formation, and may be part of a signaling pathway regulating entry into female development. However, further work is needed to characterize how these kinases function during protoperithecia formation and maturation.

### Category #3: Transcription factors

Table 3 lists the 10 transcription factor genes that we have identified as being needed for female development. Although not listed in Table 3, the genes at the two mating alleles encode transcription factors that have been well characterized and are known to play critical roles in regulating transcriptional activity during female development [81]. In *P. anserina*, a group of ten HMG-box proteins, including the mating type proteins, have been shown to function in directing perithecial development [82], [83]. Except for *pacC*, all of the transcription factors in Table 3 have been previously identified as being involved in female development by others [84]. The *asm-1* (NCU01414) transcription factor was previously identified by Aramayo et al. [85] as being needed for protoperithecium development. The *rco-1* (NCU06205), *rcm-1* (NCU06842) and *adv-1* (NCU07392) genes had been previously identified as encoding transcription factors needed for cell-to-cell fusion [35]. The *rco-1* (NCU06205) and *rcm-1* (NCU06842) genes are homologs of the *S. cerevisiae* SSN6 and TUP1genes, which encode the subunits of a general dimeric transcription factor. RCO-1 and RCM-1 have been previously identified as being needed for female development [86]. ADV-1 is a homolog of the *S. macrospora* PRO-1 protein, which has been shown to be needed for female development [87], [88]. The *ada-1* (NCU00499) and *adv-1* (NCU07392) genes were identified as transcription factors needed for both asexual and sexual development [84], and we verified their importance for female development by complementation experiments. The *fmf-1* (NCU09387) gene was previously characterized by Iyer et al. [89] as a homolog of the *S. pombe* Ste11p, a transcription factor involved in the expression of genes involved in pheromone signaling. Hutchinson and Glass [90] previously reported that the *fsd-1* (NCU09915) gene was needed for the transition from protoperithecium to a mature perithecium. The *pp-1* (NCU00340) and *pacC* (NCU00090) genes encode transcription factors that are known to function in the MAK-2 and PACC signal transcription pathways [51], [52], [91], [92]. In addition to these transcription factors that had been previously identified and characterized in *N. crassa*, one additional transcription factor for female development, MCM-1 (NCU07430) is listed in Table 3. The deletion mutant for *mcm-1* is not found in the first 119 plates of the library, but a *mcm-1* mutant has been characterized in the closely related *S. macrospora* and shown to be unable to produce perithecia [93], [94]. The *S. macrospora* MCM-1 has been shown to associate with a protein encoded by the mating type locus gene, and to regulate gene expression during perithecium development [93].

### Category #4: Autophagy genes

The identification of 10 genes in our screening and co-segregation experiments that are required for autophagy (Table 4), clearly demonstrates that autophagy is a required activity during female development. Previous work from the Pöggeler laboratory has demonstrated the importance of autophagy during *S. macrospora* female development [95]–[98]. The *atg-4*, *atg-7*, *atg-8*, *vps-34* and *vps15* genes have each been shown to be required for perithecial development in *S. macrospora*. Autophagy has been studied in *Magnaporthe oryzae*, where it has been shown to be important for asexual development, perithecial development, and appressorium formation [99], [100]. In *Aspergillus oryzae*, the deletion of autophagy genes was shown to affect the conidiation process [101]. Complementation experiments with 2 of our autophagy genes, *atg-3* and *atg-8*, verified that autophagy was required for the development of *N. crassa* protoperithecia. Some of our autophagy mutants were initially identified as being protoperithecia-defective while others were identified as being perithecia-defective. However, careful examination of the autophagy mutants shows that these mutants can initiate female development (produce ascogonia) and make a few small protoperithecia. Thus, the autophagy mutants might be best described as being able to initiate protoperithecium development, but unable to fully support subsequent female development. The protoperithecia grafting experiments demonstrate that autophagy is required within the vegetative hyphal network. We found that fertilized autophagy mutant perithecia were able to complete female development and produced ascospores when grafted onto a wild type host. We conclude that the autophagy mutants are affected in female development because the vegetative hyphal network is unable to provide an adequate supply of nutrients to the developing female.

### Category #5: Miscellaneous genes

There were 10 genes that are required for female development which didn’t fall into one of the 4 categories discussed above (Table 5). Among the miscellaneous genes we identified as being needed for female development were 3 genes that were annotated as encoding enzymes that are likely to function in melanin biosynthesis. Tyrosinase, which is encoded by the T gene (NCU00776), catalyzes the formation of melanin from dihydroxyphenylalanine (DOPA), and has been previously shown to be needed for protoperithecia formation [102]. In addition to tyrosinase, the *per-1* gene (NCU03584), which encodes a polyketide synthase that is probably needed for the production of melanin from dihydroxynapthalene (DHN), is needed for perithecium melanization. The Δ*per-1* mutant has an unmelanized perithecia phenotype [31]. We also found that an aldo-keto reductase gene (NCU01703) was needed for female development. The aldo-keto reductase may function in the pathway with the polyketide synthase for the production of DHN melanin. A polyketide synthase gene cluster has also been shown to be important for *S. macrospora* perithecial development [103], [104].

Table 5 lists 7 genes that were annotated as encoding “hypothetical proteins” or “conserved hypothetical proteins” in the *N. crassa* genome site at the Broad Institute website. These genes have been verified by complementation as being needed for female development (Table 5). We have named these genes as *fem* genes to designate that they are required for female development. Some of the encoded hypothetical proteins are highly conserved in the genomes of the filamentous fungi, suggesting that the genes play important roles in the life cycles of the filamentous fungi. As mentioned in the ascogonia formation section, *fem-1*, *fem-4*, *fem-5*, *fem-6*, and *fem-7* may function with the *div-4*, *stk-16*, *stk-22* and *stk-47* kinases in a signal transduction pathway regulating the choice between asexual and sexual developmental programs. As the annotation of the *N. crassa* genome improves and further research is done, we hope that we will be able to identify functions for many of these conserved hypothetical proteins.

## Conclusions

Our research demonstrates that a rather large number of signal transduction pathways and transcription factors are required to regulate female development (Tables 1, 2 and 3). Our analysis, in conjunction with other previously published studies, points to the involvement of at least nine different signal transduction pathways regulating female development. These are: 1) the MIK-1/MEK-1/MAK-1 cell wall integrity MAP kinase pathway; 2) the HAM-2/HAM-3/HAM-4/MOB-3 striatin pathway involved in making a complex on the nuclear envelope and in directing the movement of MAK-1 into the nucleus in a MAK-2 dependent manner; 3) the NRC-1/MEK-2/MAK-2 hyphal growth MAP kinase pathway; 4) the OS-2/OS-4/OS-5 osmotic stress MAP kinase pathway; 5) the NOX-1/NOR-1 superoxide pathway; 6) the PRE-1/PRE-2 pheromone signal transduction pathway; 7) the septation initation network (SIN), 8) a calcium signaling pathway, and 9) the PACC signal transduction pathway, which functions during the induction of protoperithecium development and during the maturation of the perithecium. Our experiments suggest that there is a signaling pathway that regulates the choice between sexual development and asexual development, and points to several genes that are likely to function in the pathway. We also found that chromatin remodeling was needed for female developments. Our experiments highlighted the role of autophagy and cell-to-cell fusion in the neighboring vegetative hyphae, which functions to provide nutrients to the developing female. What is clear from the analysis is that female development requires the coordinated activities of a number of signaling pathways.

## Materials and Methods

### Strains and growth conditions

Strains were routinely grown on Vogel’s minimal medium with 2% sucrose or on synthetic crossing medium with 0.5% sucrose [22]. The single gene deletion library was obtained from the Fungal Genetics Stock Center (Kansas City, MO). The *ΔpacC* mutant was a kind gift from Dr. Maria Bertolini (Sao Paulo, Brazil) [92]. To provide the strains needed for the complementation experiments with the pBM60/pBM61 vector system, deletion mutants were mated with a *his-3* mutant of the opposite mating type (*his-3, mat-*A FGSC#6103 or a *his-3, mat-*a isolate obtained by mating FGSC#6103 with wild-type *mat-a*) and *his-3* isolates containing the deletion mutation were isolated from among the progeny. These mating experiments were carried out as described in Davis and DeSerres [22].

To determine whether mutant isolates could form ascogonia, cellophane filters overlaid on synthetic crossing medium (without carbon/energy supplementation) were inoculated with conidia. Two to three days post-inoculum, pieces of cellophane were cut from the filters and the presence of ascogonia was assessed by observation under a compound microscope at 200 X magnification. Ascogonia formation was also assessed by inoculating synthetic crossing medium containing 0.1% sucrose with conidia and allows the cells to grow for one to three days. Agar samples were then removed from the culture and examined for ascogonia under the compound microscope.

### Screening the library

A screening procedure was used to identify mutants in the *Neurospora* deletion library that were defective in the formation of protoperithecia or for their subsequent development into mature perithecia. Plates 1 through 119 of the library contain approximately 10,000 haploid deletion mutants. Each of these mutants was individually inoculated into a glass test tube (16 × 100 mm) containing a 3 ml synthetic crossing medium agar slant. The isolates were allowed to grow for 10 days at room temperature, and examined for the presence of protoperithecia under the dissecting microscope. Protoperithecia were readily observed on the glass test tube near the edge of the synthetic crossing medium (Figure 1). After screening for protoperithecia formation, we added approximately 500 µl of water containing conidia of both *mat-*A and *mat-*a mating types to each of the slants to fertilize the protoperithecia and induce perithecium development. The slants were visually screened for the development of melanized perithecia three to four days after adding the conidial suspension. The haploid mutants isolated by the screening procedure would be considered as maternal or female developmental mutants. Most of the deletion mutants were tested for complementation in heterokaryons, and the deletion mutations found to be recessive.

### Co-segregation analysis

Many of the isolates in the *N. crassa* single gene deletion library have mutations in addition to the targeted deletion [35]. To help determine if the deletion mutations were responsible for the protoperithecium-defective and perithecium-defective phenotypes observed in the screening procedure, the mutants were subjected to a co-segregation analysis. The mutants were mated with a wild type isolate of the opposite mating type and ascospore progeny from these matings were collected [22]. Because all of the mutants were defective in female development, the mutant isolates were used as the conidial (male) partners in these matings. Ascospores from each of the matings were activated and 24 single ascospore progeny were isolated using standard procedures [22]. Each of the progeny were tested for hygromycin resistance and for the ability to form protoperithecia or perithecia with the same procedure used in the initial screening. The deletion mutations are marked by the insertion of the hygromycin resistance cassette in replacement of the deleted gene [84]. The co-segregation of hygromycin resistance with the mutant phenotype was taken as preliminary evidence that the deletion mutation was responsible for the mutant phenotype. The mutant phenotype was ascribed to a mutation other than the deletion in those cases where the mutant phenotype did not co-segregate with hygromycin resistance.

Many of the deletion mutations are represented by two isolates in the single gene deletion library, one of each mating type. In screening the library for protoperithecia-defective and perithecia-defective mutants, we found several cases where only one of the two deletion isolates in the library was defective in female development. Prior experience doing co-segregation analysis with the library showed that in such cases, the mutant phenotype invariably does not co-segregate with the deletion mutation [35]. Thus, the phenotype was assumed to be due to a mutation other than the deletion in those cases where the library contained two isolates and only one of the isolates had a mutant phenotype.

### Cell-to-cell fusion assays

A number of the previously characterized cell-to-cell fusion (anastomosis) mutants have been previously shown to be affected in female development [32], [35], [42], [45], [105]. To identify cell-to-cell fusion mutants among the mutants affected in female development, the mutants were individually tested for the ability to participate in cell-to-cell fusion with the conidial anastomosis tube fusion (CAT fusion) assay as previously described by Fu et al. [35]. The CAT fusion assay was originally described and developed by Roca et al. [40].

### Perithicia grafting experiments

The primary purpose of the protoperithecia grafting experiments was to determine whether a mutant vegetative hyphal network could support the development of transplanted wild type perithecia. A *P. anserina* perithecia grafting technique, which consists of transferring perithecia directly onto a host hyphal network has been previously described [106], [107]. In using this technique with *N. crassa*, we found that complementation between a wild type graft and a mutant host resulted in the production of multiple perithecia at the graft site. It was very difficult to differentiate between the grafted perithecia and perithecia produced by complementation within the mutant host hyphae. We therefore used a modification of the *Podospora* grafting techniques to evaluate *N. crassa* female development. To perform the grafting experiments, mutant isolates were grown on an agar plates containing SCM with 0.5% sucrose for ten days to provide “hosts” for the grafted perithecia. Wild type isolates were grown for 10 days at room temperature on a cellophane filters that had been overlaid on Petri dishes containing SCM with 0.5% sucrose agar medium. An abundance of wild type protoperithecia developed on the cellophane. A conidial suspension of the opposite mating type was then used to fertilize these wild type protoperithecia. Twenty four hours after fertilization, small pieces of cellophane (approximately 0.5 cm squares) with fertilized “graft” perithecia were cut from the filter with a sterilized new razor blade and transferred onto the Petri dishes containing mutant “host” vegetative hyphae. Three or four small pieces of cellophane containing fertilized wild type perithecia were transferred to each “host” Petri dish (Figure 2). The ability of the mutant vegetative hyphae to support development of the “graft’ wild type fertilized perithecium was determined by whether or not the perithecia on the cellophane filter ripened and shot ascospores.

In doing these grafting experiments, we determined that the host and graft had to be of the same mating type in order for the host to support perithecial development in the graft, suggesting that the vegetative incompatibility phenomenon was operative during the grafting experiments. This is in contrast with the *P. anserina* grafting experiments, in which hosts of either mating type could sustain the development of the grafted perithecia [106].

The perithecia grafting experiments allowed us to evaluate two different aspects of female development. First and foremost, they allowed us to determine whether a mutant host vegetative hyphal network could support the development of fertilized graft perithecia of the same mating type. Second, we found that grafted wild type perithecia could induce protoperithecia formation in a vegetative hyphal network of some of our mutants.

### Cloning, complementation, and RIP analysis

Complementation experiments were used to definitely demonstrate that a gene identified in the deletion library was required for female development. These complementation experiments were carried out with the pBM60/pBM61 cloning system as previously described [35]. To test for complementation, PCR primers were used to amplify the putative female development genes along with approximately 1500 base pairs of upstream sequence and 500 base pairs of downstream sequence, and the genes were cloned into the pBM60 and pBM61 vectors [108]. The primers were designed so that they contained restriction enzyme sites that allowed for the cloning of the genes into a multicloning site in the plasmids. The primers used in cloning the PACC pathway genes are given in Table S3. The pBM60 and pBM61 vectors are designed for the targeted insertion of plasmid sequences into the intergenic region downstream of the *his-3* locus. pBM60 or pBM61-derived plasmids for each of the cloned genes were used to transform an isolate having the gene deletion in a *his-3* background. Insertion of the plasmid sequences by homologous recombination generated a wild-type copy of the *his-3* gene, and allowed for the isolation of the transformant. Several transformants for each of the cloned genes were isolated and tested for the ability to make protoperithecia or perithecia on 3 ml slants of synthetic crossing medium. The ability of the cloned wild type gene to complement the deletion mutation was taken as definite proof that the gene was needed for female development.

For those mutants that did not produce conidia, the cell type used in the transformation experiments for the complementation analysis, we carried out RIP (repeat induced point mutation) analyses to verify that the mutant phenotype was due to the mutation of the targeted gene. RIP is a phenomenon in which genes that are present in two or more copies in a haploid genome are extensively mutated during the *N. crassa* sexual cycle [109]. Both copies of a duplicated gene receive multiple C to T (G to A) mutations during the RIP process. The RIP experiments were performed by cloning genes into the pBM60 or pBM61 as described above. The plasmids were used to transform *his-3* conidia to generate a strain having two copies of the cloned gene. These transformants were then mated with a *his-3* strain of the opposite mating type to activate the RIP process. Individual *his-3* progeny with the mutant phenotype were then isolated. Being *his-3* isolates, these progeny will have a single copy of the gene in question (they don’t have the “second copy” of the gene which was targeted into the *his-3* locus during the transformation). The gene was then PCR amplified and sequenced to determine if the mutant phenotype was the result of RIP mutations within the gene.

### Constitutively activated version of PACC

A constitutively activated version of PACC was created by introducing a stop codon into the *pacC* gene. The stop codon was placed such that the encoded protein lacked the C terminal 129 amino acids. This construct was generated by using the Gibson Assembly Master Mix kit (New England BioLabs, Ipswich, MA). Primers pacC-F and pacC-activated-R (Table S3) were used to amplify the 5′UTR and *pacC* coding region through the added stop codon region. Primers pacC-activated-F and pacC-R (Table S3) were used to amplify the region beginning with the stop codon and containing 3′ UTR sequences. The two PCR products were mixed with *XbaI* and *EcoRI* digested pBM60 and the Gibson Assembly Master Mix to generate a full length *pacC* containing the early stop codon.

### Intracellular localization of GFP-tagged PALA

Two versions of GFP-tagged PALA were generated to examine the localization of PALA in wild type and mutant backgrounds. Both versions were created in the pMF272 vector [62] and contain the complete PALA coding region followed by the GFP coding sequence. The two versions differed in the promoter region used to drive expression of the protein. One of the versions contained the *ccg-1* promoter found in pMF272 to drive high level expression of PALA, while the other version contained the normal *palA* promoter. For the *palA* promoter version, PCR primers palA-GFP-F and palA-GFP-R (Table S3) were used to amplify the region from 1374 base pairs upstream of the coding region to the amino acid preceding the stop codon for PALA. The PCR primers contained restriction enzyme sites to facilitate the cloning of the amplified gene and its upstream regulatory sequence immediately preceding, and in frame with, the GFP sequences in pMF272. To construct the *ccg-1* promoter version of *pal-A*, primers pal-GFP-ccg-1-F and palA-GFP-ccg-1-R (Table S3) were used to amplify and clone the coding region of *palA* into pMF272. The pMF272 vector is designed to facilitate the insertion of the cloned sequences into the intergenic region downstream of the *his-3* gene by homologous recombination [62]. The GFP-tagged *palA* plasmid constructs were used to transform the *ΔpalA, his-3* mutant to demonstrate by complementation that the GFP-tagged PALA was fully functional. The location of the GFP-tagged PALA was assessed by fluorescence confocal microscopy. Similar GFP tagging experiments were carried out to produce GPF versions of PALC and PALH, but these GFP-tagged proteins failed to complement their deletion mutants.

### Confocal microscopy

Confocal laser scanning microscopy was performed using a Zeiss LSM 710 Confocal Microscope (Carl Zeiss, Inc., USA). Plan-Apochromat 63X/1.40 oil DIC M27 objective lens or Plan-Apochromat 40X/1.3 Oil DIC M27 objective lens were used for imaging GFP expression.

## Supporting Information

Table S1 **List of protoperithecia defective/deficient mutants where the gene deletion is likely to be causing the phenotype because of general cellular health problems.** Genes that function in general metabolic pathways needed for female development are listed along with their NCU numbers. For those genes where co-segregation and complementation experiments were done to verify that the genes were needed for female development, the information is provided in the co-segregation and complementation column. Information about the type of encoded protein and the general metabolic functions it is involved in are given in the notations and protein function columns.
(DOCX)

Table S2 **Chromatin organization genes required for female development.** The genes identified in the screening procedure as being involved in generating and remodeling chromatin are listed. The deletion mutations that were shown to co-segregate with the female developmental phenotype are noted with a “yes” in the co-segregation column. Those genes that we verified as being required for female development by complementation are noted with a “yes” in the complementation column. The designation of PP in the complementation column indicates that previously published information demonstrates that the gene is needed for female development, and the reference for the information is given in the reference information column.
(DOCX)

Table S3 **Primers used in cloning PACC pathway genes.** The primers used for PCR amplification and cloning of the PACC pathway genes are listed. The added restriction sites used for inserting the genes into pMB60 and pMB61 are underlined. The pacC-F, pacC-R, pacC-activated-F, and pacC-activated-R were used with the Gibson cloning kit to introduce a stop codon into the pacC gene.
(DOCX)

## References

[1] Poggeler S, Nowrousian M, Kuck U (2006) Fruiting-Body Development in Ascomycetes; Kues U, Fischer R, editors: Springer Berlin Heidelberg.

[2] Lord KM, Read ND (2011) Perithecium morphogenesis in Sordaria macrospora. Fungal Genet Biol 48: 388–399. 21134480 10.1016/j.fgb.2010.11.009

[3] Bistis GN, Perkins DD, Read ND (2003) Different cell types in Neurospora crassa. Fungal Genet Newsl 50: 17–19.

[4] Wang Z, Lopez-Giraldez F, Lehr N, Farre M, Common R, et al (2014) Global gene expression and focused knockout analysis reveals genes associated with fungal fruiting body development in Neurospora crassa. Eukaryot Cell 13: 154–169. 24243796 10.1128/EC.00248-13 PMC3910948

[5] Hallen HE, Huebner M, Shiu SH, Guldener U, Trail F (2007) Gene expression shifts during perithecium development in Gibberella zeae (anamorph Fusarium graminearum), with particular emphasis on ion transport proteins. Fungal Genet Biol 44: 1146–1156. 17555994 10.1016/j.fgb.2007.04.007

[6] Galagan JE, Calvo SE, Borkovich KA, Selker EU, Read ND, et al (2003) The genome sequence of the filamentous fungus Neurospora crassa. Nature 422: 859–868. 12712197 10.1038/nature01554

[7] Nowrousian M, Stajich JE, Chu M, Engh I, Espagne E, et al (2010) De novo assembly of a 40 Mb eukaryotic genome from short sequence reads: Sordaria macrospora, a model organism for fungal morphogenesis. PLoS Genet 6: e1000891. 20386741 10.1371/journal.pgen.1000891 PMC2851567

[8] Teichert I, Wolff G, Kuck U, Nowrousian M (2012) Combining laser microdissection and RNA-seq to chart the transcriptional landscape of fungal development. BMC Genomics 13: 511. 23016559 10.1186/1471-2164-13-511 PMC3472292

[9] Nowrousian M, Ringelberg C, Dunlap JC, Loros JJ, Kuck U (2005) Cross-species microarray hybridization to identify developmentally regulated genes in the filamentous fungus Sordaria macrospora. Mol Genet Genomics 273: 137–149. 15778868 10.1007/s00438-005-1118-9

[10] Johnson TE (1978) Isolation and characterization of perithecial development mutants in neurospora. Genetics 88: 27–47. 17248793 10.1093/genetics/88.1.27 PMC1213789

[11] Engh I, Nowrousian M, Kuck U (2010) Sordaria macrospora, a model organism to study fungal cellular development. Eur J Cell Biol 89: 864–872. 20739093 10.1016/j.ejcb.2010.07.002

[12] Nowrousian M, Teichert I, Masloff S, Kuck U (2012) Whole-Genome Sequencing of Sordaria macrospora Mutants Identifies Developmental Genes. G3 (Bethesda) 2: 261–270. 22384404 10.1534/g3.111.001479 PMC3284333

[13] Collopy PD, Colot HV, Park G, Ringelberg C, Crew CM, et al (2010) High-throughput construction of gene deletion cassettes for generation of Neurospora crassa knockout strains. Methods Mol Biol 638: 33–40. 20238259 10.1007/978-1-60761-611-5_3 PMC3684412

[14] Borkovich KA, Alex LA, Yarden O, Freitag M, Turner GE, et al (2004) Lessons from the genome sequence of Neurospora crassa: tracing the path from genomic blueprint to multicellular organism. Microbiol Mol Biol Rev 68: 1–108. 15007097 10.1128/MMBR.68.1.1-108.2004 PMC362109

[15] Debuchey R, Bertiaux-Lecellier V, Silar P (2010) Mating systems and sexual morphologenesis in Ascomycetes. In: Borkovich KA, Ebbole DJ, editors. Cellular and Molecular Biology of Filamentous Fungi. Washington, D.C.: ASM Press. 501–535.

[16] Searle T (1973) Life cycle of Neurospora crassa viewed by scanning electron microscopy. J Bacteriol 113: 1015–1025. 4266170 10.1128/jb.113.2.1015-1025.1973 PMC285320

[17] Harris JL, Howe HB, Roth IL (1975) Scanning electron microscopy of surface and internal features of developing perithecia of *Neurospora crassa* . J Bacteriol 122: 1239–1246. 125266 10.1128/jb.122.3.1239-1246.1975 PMC246181

[18] Read ND (1983) A scanning electron microscopic study of the external features of perithecium development in *Sordaria macrospora.* . Canad J Bot 61: 3217–3229.

[19] Mai SH (1976) Morphological studies in Podospora anserina. Amer J Bot 63: 821–825.

[20] Guenther JC, Trail F (2005) The development and differentiation of Gibberella zeae (anamorph: Fusarium graminearum) during colonization of wheat. Mycologia 97: 229–237. 16389974 10.3852/mycologia.97.1.229

[21] Trail F, Common R (2000) Perithecial development by Gibberella zeae: a light microscopy study. Mycologia 92: 130–138.

[22] Davis RH, DeSerres FJ (1970) Genetic and microbiological research techniques for *Neurospora crassa* . Meth Enzymol 27: 79–143.

[23] Raju NB (1992) Genetic control of the sexual cycle of Neurospora. Mycol Res 96: 241–262.

[24] Kim H, Borkovich KA (2006) Pheromones are essential for male fertility and sufficient to direct chemotropic polarized growth of trichogynes during mating in Neurospora crassa. Eukaryot Cell 5: 544–554. 16524909 10.1128/EC.5.3.544-554.2006 PMC1398069

[25] Kim H, Borkovich KA (2004) A pheromone receptor gene, pre-1, is essential for mating type-specific directional growth and fusion of trichogynes and female fertility in Neurospora crassa. Mol Microbiol 52: 1781–1798. 15186425 10.1111/j.1365-2958.2004.04096.x

[26] Kim H, Metzenberg RL, Nelson MA (2002) Multiple functions of mfa-1, a putative pheromone precursor gene of Neurospora crassa. Eukaryot Cell 1: 987–999. 12477799 10.1128/EC.1.6.987-999.2002 PMC138756

[27] Kim H, Wright SJ, Park G, Ouyang S, Krystofova S, et al (2012) Roles for receptors, pheromones, G proteins, and mating type genes during sexual reproduction in Neurospora crassa. Genetics 190: 1389–1404. 22298702 10.1534/genetics.111.136358 PMC3316651

[28] Vigfusson NV, Weijer J (1972) Sexuality in Neurospora crassa. II. Genes affecting the sexual development cycle. Genet Res 19: 205–211. 4263084 10.1017/s0016672300014476

[29] Tan ST, Ho CC (1970) A gene controlling the early development of protoperithecium in *Neurospora crassa* . Mol Gen Genet 107: 158–161. 4938066 10.1007/BF00333631

[30] Mylyk OM, Threlkeld SF (1974) A genetic study of female sterility in Neurospora crassa. Genet Res 24: 91–102. 4279838 10.1017/s001667230001510x

[31] McCluskey K, Wiest AE, Grigoriev IV, Lipzen A, Martin J, et al (2011) Rediscovery by Whole Genome Sequencing: Classical Mutations and Genome Polymorphisms in Neurospora crassa. G3 (Bethesda) 1: 303–316. 22384341 10.1534/g3.111.000307 PMC3276140

[32] Fleissner A, Sarkar S, Jacobson DJ, Roca MG, Read ND, et al (2005) The so locus is required for vegetative cell fusion and postfertilization events in Neurospora crassa. Eukaryot Cell 4: 920–930. 15879526 10.1128/EC.4.5.920-930.2005 PMC1140088

[33] Howe HB, Bensen EW (1974) A perithecial color mutant of *Neurospora crassa* . Mol Gen Genet 131: 79–83. 4277338 10.1007/BF00269389

[34] Horowitz NH, Fling M, MacLeod H, Sueoka N (1961) A genetic study of two structural forms of tyrosinase in *Neurospora.* . Genetics 46: 1015–1024. 13715943 10.1093/genetics/46.8.1015 PMC1210246

[35] Fu C, Iyer P, Herkal A, Abdullah J, Stout A, et al (2011) Identification and characterization of genes required for cell-to-cell fusion in Neurospora crassa. Eukaryot Cell 10: 1100–1109. 21666072 10.1128/EC.05003-11 PMC3165452

[36] Adhvaryu KK, Morris SA, Strahl BD, Selker EU (2005) Methylation of histone H3 lysine 36 is required for normal development in Neurospora crassa. Eukaryot Cell 4: 1455–1464. 16087750 10.1128/EC.4.8.1455-1464.2005 PMC1214527

[37] Brenna A, Grimaldi B, Filetici P, Ballario P (2012) Physical association of the WC-1 photoreceptor and the histone acetyltransferase NGF-1 is required for blue light signal transduction in Neurospora crassa. Mol Biol Cell 23: 3863–3872. 22875992 10.1091/mbc.E12-02-0142 PMC3459862

[38] Lewis ZA, Adhvaryu KK, Honda S, Shiver AL, Knip M, et al (2010) DNA methylation and normal chromosome behavior in Neurospora depend on five components of a histone methyltransferase complex, DCDC. PLoS Genet 6: e1001196. 21079689 10.1371/journal.pgen.1001196 PMC2973830

[39] Gesing S, Schindler D, Franzel B, Wolters D, Nowrousian M (2012) The histone chaperone ASF1 is essential for sexual development in the filamentous fungus Sordaria macrospora. Mol Microbiol 84: 748–765. 22463819 10.1111/j.1365-2958.2012.08058.x

[40] Roca MG, Arlt J, Jeffree CE, Read ND (2005) Cell biology of conidial anastomosis tubes in Neurospora crassa. Eukaryot Cell 4: 911–919. 15879525 10.1128/EC.4.5.911-919.2005 PMC1140100

[41] Maerz S, Dettmann A, Ziv C, Liu Y, Valerius O, et al (2009) Two NDR kinase-MOB complexes function as distinct modules during septum formation and tip extension in Neurospora crassa. Mol Microbiol 74: 707–723. 19788544 10.1111/j.1365-2958.2009.06896.x PMC4617822

[42] Xiang Q, Rasmussen C, Glass NL (2002) The ham-2 locus, encoding a putative transmembrane protein, is required for hyphal fusion in Neurospora crassa. Genetics 160: 169–180. 11805054 10.1093/genetics/160.1.169 PMC1461943

[43] Dettmann A, Heilig Y, Ludwig S, Schmitt K, Illgen J, et al (2013) HAM-2 and HAM-3 are central for the assembly of the Neurospora STRIPAK complex at the nuclear envelope and regulate nuclear accumulation of the MAP kinase MAK-1 in a MAK-2-dependent manner. Mol Microbiol 90: 796–812. 24028079 10.1111/mmi.12399

[44] Fleissner A, Leeder AC, Roca MG, Read ND, Glass NL (2009) Oscillatory recruitment of signaling proteins to cell tips promotes coordinated behavior during cell fusion. Proc Natl Acad Sci U S A 106: 19387–19392. 19884508 10.1073/pnas.0907039106 PMC2780775

[45] Lichius A, Lord KM, Jeffree CE, Oborny R, Boonyarungsrit P, et al (2012) Importance of MAP kinases during protoperithecial morphogenesis in Neurospora crassa. PLoS One 7: e42565. 22900028 10.1371/journal.pone.0042565 PMC3416862

[46] Kothe GO, Free SJ (1998) Calcineurin subunit B is required for normal vegetative growth in Neurospora crassa. Fungal Genet Biol 23: 248–258. 9680955 10.1006/fgbi.1998.1037

[47] Fu C, Ao J, Dettmann A, Seiler S, Free SJ (2014) Characterization of the Neurospora crassa cell fusion proteins, HAM-6, HAM-7, HAM-8, HAM-9, HAM-10, AMPH-1, and WHI-2. PLoS One in press. 10.1371/journal.pone.0107773 PMC4184795 25279949

[48] Park G, Servin JA, Turner GE, Altamirano L, Colot HV, et al (2011) Global analysis of serine-threonine protein kinase genes in Neurospora crassa. Eukaryot Cell 10: 1553–1564. 21965514 10.1128/EC.05140-11 PMC3209061

[49] Arst HN, Penalva MA (2003) pH regulation in Aspergillus and parallels with higher eukaryotic regulatory systems. Trends Genet 19: 224–231. 12683976 10.1016/s0168-9525(03)00052-0

[50] Penalva MA, Arst HN Jr (2002) Regulation of gene expression by ambient pH in filamentous fungi and yeasts. Microbiol Mol Biol Rev 66: 426–446, table of contents. 10.1128/MMBR.66.3.426-446.2002 PMC120796 12208998

[51] Penalva MA, Tilburn J, Bignell E, Arst HN Jr (2008) Ambient pH gene regulation in fungi: making connections. Trends Microbiol 16: 291–300. 18457952 10.1016/j.tim.2008.03.006

[52] Mitchell AP (2008) A VAST staging area for regulatory proteins. Proc Natl Acad Sci U S A 105: 7111–7112. 18474868 10.1073/pnas.0803384105 PMC2438211

[53] Lamb TM, Xu W, Diamond A, Mitchell AP (2001) Alkaline response genes of Saccharomyces cerevisiae and their relationship to the RIM101 pathway. J Biol Chem 276: 1850–1856. 11050096 10.1074/jbc.M008381200

[54] Diez E, Alvaro J, Espeso EA, Rainbow L, Suarez T, et al (2002) Activation of the Aspergillus PacC zinc finger transcription factor requires two proteolytic steps. EMBO J 21: 1350–1359. 11889040 10.1093/emboj/21.6.1350 PMC125927

[55] Li W, Mitchell AP (1997) Proteolytic activation of Rim1p, a positive regulator of yeast sporulation and invasive growth. Genetics 145: 63–73. 9017390 10.1093/genetics/145.1.63 PMC1207785

[56] Ramon AM, Porta A, Fonzi WA (1999) Effect of environmental pH on morphological development of Candida albicans is mediated via the PacC-related transcription factor encoded by PRR2. J Bacteriol 181: 7524–7530. 10601210 10.1128/jb.181.24.7524-7530.1999 PMC94210

[57] Su SS, Mitchell AP (1993) Identification of functionally related genes that stimulate early meiotic gene expression in yeast. Genetics 133: 67–77. 8417990 10.1093/genetics/133.1.67 PMC1205299

[58] Xu W, Smith FJ Jr, Subaran R, Mitchell AP (2004) Multivesicular body-ESCRT components function in pH response regulation in Saccharomyces cerevisiae and Candida albicans. Mol Biol Cell 15: 5528–5537. 15371534 10.1091/mbc.E04-08-0666 PMC532031

[59] Davis D, Wilson RB, Mitchell AP (2000) RIM101-dependent and-independent pathways govern pH responses in Candida albicans. Mol Cell Biol 20: 971–978. 10629054 10.1128/mcb.20.3.971-978.2000 PMC85214

[60] Li M, Martin SJ, Bruno VM, Mitchell AP, Davis DA (2004) Candida albicans Rim13p, a protease required for Rim101p processing at acidic and alkaline pHs. Eukaryot Cell 3: 741–751. 15189995 10.1128/EC.3.3.741-751.2004 PMC420141

[61] Rollins JA (2003) The Sclerotinia sclerotiorum pac1 gene is required for sclerotial development and virulence. Mol Plant Microbe Interact 16: 785–795. 12971602 10.1094/MPMI.2003.16.9.785

[62] Bowman BJ, Draskovic M, Freitag M, Bowman EJ (2009) Structure and distribution of organelles and cellular location of calcium transporters in Neurospora crassa. Eukaryot Cell 8: 1845–1855. 19801418 10.1128/EC.00174-09 PMC2794220

[63] Pandey A, Roca MG, Read ND, Glass NL (2004) Role of a mitogen-activated protein kinase pathway during conidial germination and hyphal fusion in Neurospora crassa. Eukaryot Cell 3: 348–358. 15075265 10.1128/EC.3.2.348-358.2004 PMC387641

[64] Kothe GO, Free SJ (1998) The isolation and characterization of nrc-1 and nrc-2, two genes encoding protein kinases that control growth and development in Neurospora crassa. Genetics 149: 117–130. 9584090 10.1093/genetics/149.1.117 PMC1460147

[65] Kaida D, Yashiroda H, Toh-e A, Kikuchi Y (2002) Yeast Whi2 and Psr1-phosphatase form a complex and regulate STRE-mediated gene expression. Genes Cells 7: 543–552. 12090248 10.1046/j.1365-2443.2002.00538.x

[66] Leadsham JE, Miller K, Ayscough KR, Colombo S, Martegani E, et al (2009) Whi2p links nutritional sensing to actin-dependent Ras-cAMP-PKA regulation and apoptosis in yeast. J Cell Sci 122: 706–715. 19208759 10.1242/jcs.042424 PMC2720921

[67] Mendl N, Occhipinti A, Muller M, Wild P, Dikic I, et al (2011) Mitophagy in yeast is independent of mitochondrial fission and requires the stress response gene WHI2. J Cell Sci 124: 1339–1350. 21429936 10.1242/jcs.076406

[68] Muller M, Reichert AS (2011) Mitophagy, mitochondrial dynamics and the general stress response in yeast. Biochem Soc Trans 39: 1514–1519. 21936844 10.1042/BST0391514

[69] Cano-Dominguez N, Alvarez-Delfin K, Hansberg W, Aguirre J (2008) NADPH oxidases NOX-1 and NOX-2 require the regulatory subunit NOR-1 to control cell differentiation and growth in Neurospora crassa. Eukaryot Cell 7: 1352–1361. 18567788 10.1128/EC.00137-08 PMC2519770

[70] Aguirre J, Rios-Momberg M, Hewitt D, Hansberg W (2005) Reactive oxygen species and development in microbial eukaryotes. Trends Microbiol 13: 111–118. 15737729 10.1016/j.tim.2005.01.007

[71] Takemoto D, Tanaka A, Scott B (2007) NADPH oxidases in fungi: diverse roles of reactive oxygen species in fungal cellular differentiation. Fungal Genet Biol 44: 1065–1076. 17560148 10.1016/j.fgb.2007.04.011

[72] Dirschnabel DE, Nowrousian M, Cano-Dominguez N, Aguirre J, Teichert I, et al (2014) New Insights Into the Roles of NADPH Oxidases in Sexual Development and Ascospore Germination in Sordaria macrospora. Genetics 196: 729–744. 24407906 10.1534/genetics.113.159368 PMC3948803

[73] Malagnac F, Lalucque H, Lepere G, Silar P (2004) Two NADPH oxidase isoforms are required for sexual reproduction and ascospore germination in the filamentous fungus Podospora anserina. Fungal Genet Biol 41: 982–997. 15465387 10.1016/j.fgb.2004.07.008

[74] Mayrhofer S, Weber JM, Poggeler S (2006) Pheromones and pheromone receptors are required for proper sexual development in the homothallic ascomycete Sordaria macrospora. Genetics 172: 1521–1533. 16387884 10.1534/genetics.105.047381 PMC1456310

[75] Bloemendal S, Lord KM, Rech C, Hoff B, Engh I, et al (2010) A mutant defective in sexual development produces aseptate ascogonia. Eukaryot Cell 9: 1856–1866. 20952581 10.1128/EC.00186-10 PMC3008273

[76] Heilig Y, Schmitt K, Seiler S (2013) Phospho-regulation of the Neurospora crassa septation initiation network. PLoS One 8: e79464. 24205386 10.1371/journal.pone.0079464 PMC3804505

[77] Justa-Schuch D, Heilig Y, Richthammer C, Seiler S (2010) Septum formation is regulated by the RHO4-specific exchange factors BUD3 and RGF3 and by the landmark protein BUD4 in Neurospora crassa. Mol Microbiol 76: 220–235. 20199606 10.1111/j.1365-2958.2010.07093.x

[78] Heilig Y, Dettmann A, Mourino-Perez RR, Schmitt K, Valerius O, et al (2014) Proper actin ring formation and septum constriction requires coordinated regulation of SIN and MOR pathways through the germinal centre kinase MST-1. PLoS Genet 10: e1004306. 24762679 10.1371/journal.pgen.1004306 PMC3998894

[79] Rasmussen CG, Glass NL (2005) A Rho-type GTPase, rho-4, is required for septation in Neurospora crassa. Eukaryot Cell 4: 1913–1925. 16278458 10.1128/EC.4.11.1913-1925.2005 PMC1287859

[80] Riquelme M, Yarden O, Bartnicki-Garcia S, Bowman B, Castro-Longoria E, et al (2011) Architecture and development of the Neurospora crassa hypha – a model cell for polarized growth. Fungal Biol 115: 446–474. 21640311 10.1016/j.funbio.2011.02.008

[81] Debuchy R, Berteaux-Lecellier V, Silar P (2010) Mating Systems and Sexual Morphogenesis in Ascomycetes. In: Borkovich KA, Ebbole DJ, editors. Cellular and Molecular Biology of Filamentous Fungi. Washington DC: ASM Press. 501–536.

[82] Benkhali JA, Coppin E, Brun S, Peraza-Reyes L, Martin T, et al.. (2013) A Network of HMG-box Transcription Factors Regulates Sexual Cycle in the Fungus Podospora anserina. Plos Genetics 9. 10.1371/journal.pgen.1003642 PMC3730723 23935511

[83] Coppin E, Berteaux-Lecellier V, Bidard F, Brun S, Ruprich-Robert G, et al (2012) Systematic deletion of homeobox genes in Podospora anserina uncovers their roles in shaping the fruiting body. PLoS One 7: e37488. 22662159 10.1371/journal.pone.0037488 PMC3360767

[84] Colot HV, Park G, Turner GE, Ringelberg C, Crew CM, et al (2006) A high-throughput gene knockout procedure for Neurospora reveals functions for multiple transcription factors. Proc Natl Acad Sci U S A 103: 10352–10357. 16801547 10.1073/pnas.0601456103 PMC1482798

[85] Aramayo R, Peleg Y, Addison R, Metzenberg R (1996) Asm-1+, a Neurospora crassa gene related to transcriptional regulators of fungal development. Genetics 144: 991–1003. 8913744 10.1093/genetics/144.3.991 PMC1207638

[86] Aldabbous MS, Roca MG, Stout A, Huang IC, Read ND, et al (2010) The ham-5, rcm-1 and rco-1 genes regulate hyphal fusion in Neurospora crassa. Microbiology 156: 2621–2629. 20522492 10.1099/mic.0.040147-0 PMC3068686

[87] Masloff S, Poggeler S, Kuck U (1999) The pro1(+) gene from Sordaria macrospora encodes a C6 zinc finger transcription factor required for fruiting body development. Genetics 152: 191–199. 10224253 10.1093/genetics/152.1.191 PMC1460585

[88] Masloff S, Jacobsen S, Poggeler S, Kuck U (2002) Functional analysis of the C6 zinc finger gene pro1 involved in fungal sexual development. Fungal Genet Biol 36: 107–116. 12081464 10.1016/S1087-1845(02)00010-5

[89] Iyer SV, Ramakrishnan M, Kasbekar DP (2009) Neurospora crassa fmf-1 encodes the homologue of the Schizosaccharomyces pombe Ste11p regulator of sexual development. J Genet 88: 33–39. 19417542 10.1007/s12041-009-0005-2

[90] Hutchison EA, Glass NL (2010) Meiotic regulators Ndt80 and ime2 have different roles in Saccharomyces and Neurospora. Genetics 185: 1271–1282. 20519745 10.1534/genetics.110.117184 PMC2927755

[91] Leeder AC, Jonkers W, Li J, Glass NL (2013) Early colony establishment in Neurospora crassa requires a MAP kinase regulatory network. Genetics 195: 883–898. 24037267 10.1534/genetics.113.156984 PMC3813871

[92] Cupertino FB, Freitas FZ, de Paula RM, Bertolini MC (2012) Ambient pH controls glycogen levels by regulating glycogen synthase gene expression in Neurospora crassa. New insights into the pH signaling pathway. PLoS One 7: e44258. 22952943 10.1371/journal.pone.0044258 PMC3432076

[93] Nolting N, Poggeler S (2006) A MADS box protein interacts with a mating-type protein and is required for fruiting body development in the homothallic ascomycete Sordaria macrospora. Eukaryot Cell 5: 1043–1056. 16835449 10.1128/EC.00086-06 PMC1489284

[94] Nolting N, Poggeler S (2006) A STE12 homologue of the homothallic ascomycete Sordaria macrospora interacts with the MADS box protein MCM1 and is required for ascosporogenesis. Mol Microbiol 62: 853–868. 16999832 10.1111/j.1365-2958.2006.05415.x

[95] Voigt O, Herzog B, Jakobshagen A, Poggeler S (2013) bZIP transcription factor SmJLB1 regulates autophagy-related genes Smatg8 and Smatg4 and is required for fruiting-body development and vegetative growth in Sordaria macrospora. Fungal Genet Biol 61: 50–60. 24095659 10.1016/j.fgb.2013.09.006

[96] Voigt O, Herzog B, Jakobshagen A, Poggeler S (2014) Autophagic kinases SmVPS34 and SmVPS15 are required for viability in the filamentous ascomycete Sordaria macrospora. Microbiol Res 169: 128–138. 23953726 10.1016/j.micres.2013.07.012

[97] Voigt O, Poggeler S (2013) Autophagy genes Smatg8 and Smatg4 are required for fruiting-body development, vegetative growth and ascospore germination in the filamentous ascomycete Sordaria macrospora. Autophagy 9: 33–49. 23064313 10.4161/auto.22398 PMC3542216

[98] Nolting N, Bernhards Y, Poggeler S (2009) SmATG7 is required for viability in the homothallic ascomycete Sordaria macrospora. Fungal Genet Biol 46: 531–542. 19351563 10.1016/j.fgb.2009.03.008

[99] Lin F-C, Liu X-H, Lu J-P, Liu T-B (2009) Studies on Autophagy Machinery in Magnaporthe oryzae. In: Wang G-L, Valent B, editors. Advances in Genetics, Genomics, and Control of Rice Blast Disease: Springer. 33–40.

[100] Liu TB, Liu XH, Lu JP, Zhang L, Min H, et al (2010) The cysteine protease MoAtg4 interacts with MoAtg8 and is required for differentiation and pathogenesis in Magnaporthe oryzae. Autophagy 6: 74–85. 19923912 10.4161/auto.6.1.10438

[101] Kikuma T, Kitamoto K (2011) Analysis of autophagy in Aspergillus oryzae by disruption of Aoatg13, Aoatg4, and Aoatg15 genes. FEMS Microbiol Lett 316: 61–69. 21204928 10.1111/j.1574-6968.2010.02192.x

[102] Fuentes AM, Connerton I, Free SJ (1994) Production of tyrosinase defective mutants in *Neurospora crassa* . Fungal Genet Newsl 41: 38–39.

[103] Nowrousian M (2009) A novel polyketide biosynthesis gene cluster is involved in fruiting body morphogenesis in the filamentous fungi Sordaria macrospora and Neurospora crassa. Curr Genet 55: 185–198. 19277664 10.1007/s00294-009-0236-z

[104] Schindler D, Nowrousian M (2014) The polyketide synthase gene pks4 is essential for sexual development and regulates fruiting body morphology in Sordaria macrospora. Fungal Genet Biol 68: 48–59. 24792494 10.1016/j.fgb.2014.04.008

[105] Park G, Pan S, Borkovich KA (2008) Mitogen-activated protein kinase cascade required for regulation of development and secondary metabolism in Neurospora crassa. Eukaryot Cell 7: 2113–2122. 18849472 10.1128/EC.00466-07 PMC2593188

[106] Silar P (2011) Grafting as a method for studying development in the filamentous fungus Podospora anserina. Fungal Biol 115: 793–802. 21802060 10.1016/j.funbio.2011.06.005

[107] Silar P (2014) Simple genetic tools to study fruiting body development in fungi. Open Mycol J 8: 148–155.

[108] Margolin BS, Frietag M, Selker EU (1997) Improved plasmids for gene targeting at the *his-3* locus of *Neurospora crassa* . Fungal Genet Newsl 44: 34–36.

[109] Selker EU (1999) Gene silencing: repeats that count. Cell 97: 157–160. 10219236 10.1016/s0092-8674(00)80725-4

[110] Maerz S, Ziv C, Vogt N, Helmstaedt K, Cohen N, et al (2008) The nuclear Dbf2-related kinase COT1 and the mitogen-activated protein kinases MAK1 and MAK2 genetically interact to regulate filamentous growth, hyphal fusion and sexual development in Neurospora crassa. Genetics 179: 1313–1325. 18562669 10.1534/genetics.108.089425 PMC2475735

[111] Li D, Bobrowicz P, Wilkinson HH, Ebbole DJ (2005) A mitogen-activated protein kinase pathway essential for mating and contributing to vegetative growth in Neurospora crassa. Genetics 170: 1091–1104. 15802524 10.1534/genetics.104.036772 PMC1451179

[112] Dettmann A, Illgen J, Marz S, Schurg T, Fleissner A, et al (2012) The NDR kinase scaffold HYM1/MO25 is essential for MAK2 map kinase signaling in Neurospora crassa. PLoS Genet 8: e1002950. 23028357 10.1371/journal.pgen.1002950 PMC3447951

[113] Araujo-Palomares CL, Richthammer C, Seiler S, Castro-Longoria E (2011) Functional characterization and cellular dynamics of the CDC-42 - RAC - CDC-24 module in Neurospora crassa. PLoS One 6: e27148. 22087253 10.1371/journal.pone.0027148 PMC3210136

[114] Richthammer C, Enseleit M, Sanchez-Leon E, Marz S, Heilig Y, et al (2012) RHO1 and RHO2 share partially overlapping functions in the regulation of cell wall integrity and hyphal polarity in Neurospora crassa. Mol Microbiol 85: 716–733. 22703449 10.1111/j.1365-2958.2012.08133.x

[115] Vogt N, Seiler S (2008) The RHO1-specific GTPase-activating protein LRG1 regulates polar tip growth in parallel to Ndr kinase signaling in Neurospora. Mol Biol Cell 19: 4554–4569. 18716060 10.1091/mbc.E07-12-1266 PMC2575149

[116] Engh I, Wurtz C, Witzel-Schlomp K, Zhang HY, Hoff B, et al (2007) The WW domain protein PRO40 is required for fungal fertility and associates with Woronin bodies. Eukaryot Cell 6: 831–843. 17351077 10.1128/EC.00269-06 PMC1899833

[117] Simonin AR, Rasmussen CG, Yang M, Glass NL (2010) Genes encoding a striatin-like protein (ham-3) and a forkhead associated protein (ham-4) are required for hyphal fusion in Neurospora crassa. Fungal Genet Biol 47: 855–868. 20601042 10.1016/j.fgb.2010.06.010

[118] Bloemendal S, Bernhards Y, Bartho K, Dettmann A, Voigt O, et al (2012) A homologue of the human STRIPAK complex controls sexual development in fungi. Mol Microbiol 84: 310–323. 22375702 10.1111/j.1365-2958.2012.08024.x

[119] Nowrousian M, Frank S, Koers S, Strauch P, Weitner T, et al (2007) The novel ER membrane protein PRO41 is essential for sexual development in the filamentous fungus Sordaria macrospora. Mol Microbiol 64: 923–937. 17501918 10.1111/j.1365-2958.2007.05694.x PMC3694341

[120] Maddi A, Dettman A, Fu C, Seiler S, Free SJ (2012) WSC-1 and HAM-7 are MAK-1 MAP kinase pathway sensors required for cell wall integrity and hyphal fusion in Neurospora crassa. PLoS One 7: e42374. 22879952 10.1371/journal.pone.0042374 PMC3411791

[121] Jones CA, Greer-Phillips SE, Borkovich KA (2007) The response regulator RRG-1 functions upstream of a mitogen-activated protein kinase pathway impacting asexual development, female fertility, osmotic stress, and fungicide resistance in Neurospora crassa. Mol Biol Cell 18: 2123–2136. 17392518 10.1091/mbc.E06-03-0226 PMC1877117

[122] Poggeler S (2000) Two pheromone precursor genes are transcriptionally expressed in the homothallic ascomycete Sordaria macrospora. Curr Genet 37: 403–411. 10905431 10.1007/s002940000120

[123] Ivey FD, Kays AM, Borkovich KA (2002) Shared and independent roles for a Galpha(i) protein and adenylyl cyclase in regulating development and stress responses in Neurospora crassa. Eukaryot Cell 1: 634–642. 12456011 10.1128/EC.1.4.634-642.2002 PMC118002

[124] Kamerewerd J, Jansson M, Nowrousian M, Poggeler S, Kuck U (2008) Three alpha-subunits of heterotrimeric G proteins and an adenylyl cyclase have distinct roles in fruiting body development in the homothallic fungus Sordaria macrospora. Genetics 180: 191–206. 18723884 10.1534/genetics.108.091603 PMC2535674

[125] Yang Q, Poole SI, Borkovich KA (2002) A G-protein beta subunit required for sexual and vegetative development and maintenance of normal G alpha protein levels in Neurospora crassa. Eukaryot Cell 1: 378–390. 12455986 10.1128/EC.1.3.378-390.2002 PMC118013

[126] Krystofova S, Borkovich KA (2005) The heterotrimeric G-protein subunits GNG-1 and GNB-1 form a Gbetagamma dimer required for normal female fertility, asexual development, and galpha protein levels in Neurospora crassa. Eukaryot Cell 4: 365–378. 15701799 10.1128/EC.4.2.365-378.2005 PMC549333

[127] Muller F, Kruger D, Sattlegger E, Hoffmann B, Ballario P, et al (1995) The cpc-2 gene of Neurospora crassa encodes a protein entirely composed of WD-repeat segments that is involved in general amino acid control and female fertility. Mol Gen Genet 248: 162–173. 7651339 10.1007/BF02190797

[128] Yamashiro CT, Ebbole DJ, Lee BU, Brown RE, Bourland C, et al (1996) Characterization of rco-1 of Neurospora crassa, a pleiotropic gene affecting growth and development that encodes a homolog of Tup1 of Saccharomyces cerevisiae. Mol Cell Biol 16: 6218–6228. 8887652 10.1128/mcb.16.11.6218 PMC231625

[129] Kim SR, Lee B-U (2005) Characterization of the *Neurospora crassa rcm-1* mutant. Kor J Microbiol 41: 246–254.

