# Supplementary material for: Neurospora crassa Female Development Requires the PACC and Other Signal Transduction Pathways, Transcription Factors, Chromatin Remodeling, Cell-To-Cell Fusion, and Autophagy
Source: PLoS One. 2014 Oct 21;9(10):e110603. doi: 10.1371/journal.pone.0110603 (PMC4204872; doi:10.1371/journal.pone.0110603)
Supplement: Table S1 — List of protoperithecia defective/deficient mutants where the gene deletion is likely to be causing the phenotype because of general cellular health problems. Genes that function in general metabolic pathways needed for female development are listed along with their NCU numbers. For those genes where co-segregation and complementation experiments were done to verify that the genes were needed for female development, the information is provided in the co-segregation and complementation column. Information about the type of encoded protein and the general metabolic functions it is involved in are given in the notations and protein function columns. (DOCX) [file pone.0110603.s001.docx]

**Table S1. List of protoperithecia defective/deficient mutants where the gene deletion is likely to be causing the phenotype because of general cellular health problems.**

| Gene name | NCU# | Co-segregation and complementation information | Notations | Protein function |
| --- | --- | --- | --- | --- |
| Ro-6 Dynein intermed. chain | 09142 | Verified by co-segregation and complementation | Needed for cytoskeleton and nuclear transport; Fu et al. [[35](#_ENREF_35)] | Nuclear movement |
| Ro-11 dynein/dynactin associated protein | 08560 | Verified by co-segregation and complementation | Needed for cytoskeleton and nuclear transport; Fu et al. [[35](#_ENREF_35)] | Nuclear movement |
| Conserved hypothet | 08992 | Verified by co-segregation and complementation | Cytochrome c oxidase assembly | Mito. ATP synthesis |
| Rox3 mediator complex subunit | 01475 | Co-segregation | Perithecia-defective  Dark medium | Rox3 is subunit of RNA polymerase complex |
| Nuclear pore complex protein | 00486 | Co-segregation | Complementation – this report | SAM dependent methyl transferase – darkened medium |
| Conserved hypothet | 04325 | Co-segregation | Mediator of RNA polymerase transcription subunit 1 | Perithecia-defective |
| Cytochrome c oxidase | 06145 | Co-segregation | Electron transport chain | Mito. ATP synthesis |
| Och-1 | 00609 | Verified by co-segregation and  complementation | Maddi and Free [[130](#_ENREF_130)] | Cell wall biogenesis |
| Telomeric repeat binding factor 1 | 03416 |  | Telomere binding protein | Chromosome integrity |
| Pre mRNA processing | 00158 |  | Pre-mRNA processing | mRNA synthesis |
| SRP subunit | 00635 |  | Nascent polypeptide-associated complex subunit alpha | Protein secretion |
| SRP subunit | 03148 |  | Nascent polypeptide-associated complex subunit beta | Protein secretion |
| DNA damage inducible gene 1 | 05292 | Co-segregation |  | Proteasome function |
| ubiquitin conjugation factor E4 | 03357 | Co-segregation | Darkened medium | Protein degradation |
| Hpth1 | 01216 | Co-segregation | Dark medium, sick, and semi-colonial | Ubiquitin ligase protein |
|  | 03559 |  | Ubiquinol-cytochrome c reductase complex subunit | Mito. ATP synthesis |
| Nuo21.3b | 02280 |  | NADH:ubiquinone oxidoreductase subunit | Mito. ATP synthesis |
| Mago nashi | 04405 |  | mRNA splicing protein | mRNA synthesis |
| Enoyl-CoA hydratase | 03542 |  | Enoyl-CoA hydratase | Fatty acid metabolism |
| Rtf1 | 03956 |  | DNA-binding rtf1 protein – RNA polymerase TATA binding and elongation | General transcription |
| Pkr1 | 00506 | Verified by co-segregation and complementation | ER protein – needed for assembly of vacuolar ATPase; Fu et al. [[35](#_ENREF_35)] | Vesicular trafficking |
| Cps-1 | 00911 | Verified by co-segregation and RIP | Polysaccharide synthase cps1: Fu et al. [[131](#_ENREF_131)] | Cell wall synthesis |
| Hypothetical protein | 02304 |  | ER protein needed for assembly of vacuolar ATPase | Vesicular trafficking |
| Cia84 | 01006 | Co-segregation | Complex I intermediate associated protein 84 – assembly of NADH:ubiquinone oxidoreductase | Mito. ATP synthesis |
| Hypothetical | 07494 |  | Ribosome recycling factor | Protein synthesis |
| Hypothetical | 07329 |  | Mediator complex protein – needed for RNA PolII function | General transcription |
| Actin-family protein | 03563 |  | Odd morphology, yellow/orange conidia | Cytoskeleton organization |
| Hypothetical | 02451 |  | Mitochondrial Hypoxia-response | Mito function |
| Hypothetical protein | 04032 |  | U1 zinc finger protein | RNA splicing |
| RING/Zn protein | 06882 | Verified by co-segregation and complementation | Likely component of ubiquitin ligase -dark medium | Protein degradation |
| Deoxyhypusine hydroxylase | 05252 | Co-segregation | Needed for protein initiation factor EIF5A | Protein translation |
| Thioredoxin | 09803 |  | Needed to keep cytosol in a reduced state | General protein structure |
| Hypothetical protein | 00967 |  | Fragile X domain – for ribosome assembly and telomer maintenance | Chromosome integrity |
| Hypothetical protein | 02033 |  | RNA polymerase II transcription elongation factor – dark medium | General transcription |
| Hypothetical protein | 02382 |  | Ubiquitin carboxyl-terminus hydrolase | Needed for processing and recycling of ubiquitin |
| Mmp37 | 04429 | Co-segregation | Mitochondrial import protein | Mitochondrial function |
| 60S ribosomal protein L1 | 08893 |  | Ribosomal protein L1 | Protein translation |
| AIM24 | 04645 |  | Mitochondrial biogenesis protein | Mitochondrial function |
| C-type cyclin | 04495 | Co-segregation | The cyclin may be specific to female development – mutant doesn’t have a growth defect | Cell cycle |
| Pre-MRNA splicing factor | 07069 |  | Pre-mRNA splicing factor | mRNA splicing |
| Hypothetical protein | 01303 |  | Cohesin loading factor – loads cohesion onto chromosome in G1 | Chromosome integrity |
| Por  (porin) | 04304 |  | Porin – mitochondrial outer membrane | Mitochondrial function |
| ff-1 | 01543 |  | PTAB – TFIIA topoisomerase associated protein pat1 – functions in translation- CAP binding; McCluskey et al. [[31](#_ENREF_31)] | General transcription |
| Small nuclear ribonuclear protein U | 08034 |  | Likely splicing protein | mRNA splicing |
| Mitochondrial thiamine pyrophosphate carrier | 07384 |  | Carrier for import into mitochondria | Mitochondrial function |
| Cytochrome c lyase | 08138 | Co-segregation | Needed to attach heme to cytochrome c | Mitochondrial function |
| ATP citrate lyase | 06783 |  | Needed to make acetyl-CoA for fatty acid synthesis | Fatty acid synthesis |
| Electron transport flavoprotein alpha-subunit | 08004 | Co-segregation | Needed for electron transport chain | Mitochondrial function |
| Mitochondrial DNA replication protein YHM2 | 01689 | Co-segregation | Needed for mitochondrial DNA replication | Mitochondrial function |
| Eif3e | 05889 |  | Translation initiation factor 3 subunit 6 | Protein translation |
| NADH:ubiquinone oxidoreductase 12.3 | 03093 |  | Needed for electron transport chain | Mitochondrial function |
| Ro-10 (ropy-10)  Dynein/dynactin associated protein | 10696 |  | Needed for cytoskeleton and nuclear transport | Nuclear movement |
| Ypt1 | 08477 |  | Rab-GTPase involved in trafficking from ER to Golgi | Vesicular trafficking |
