# Supplementary material for: Neurospora crassa Female Development Requires the PACC and Other Signal Transduction Pathways, Transcription Factors, Chromatin Remodeling, Cell-To-Cell Fusion, and Autophagy
Source: PLoS One. 2014 Oct 21;9(10):e110603. doi: 10.1371/journal.pone.0110603 (PMC4204872; doi:10.1371/journal.pone.0110603)
Supplement: Table S2 — Chromatin organization genes required for female development. The genes identified in the screening procedure as being involved in generating and remodeling chromatin are listed. The deletion mutations that were shown to co-segregate with the female developmental phenotype are noted with a “yes” in the co-segregation column. Those genes that we verified as being required for female development by complementation are noted with a “yes” in the complementation column. The designation of PP in the complementation column indicates that previously published information demonstrates that the gene is needed for female development, and the reference for the information is given in the reference information column. (DOCX) [file pone.0110603.s002.docx]

**Table S2. Chromatin organization genes required for female development.**

| Gene | NCU# | Co-segregation | Complementation | Reference Information |
| --- | --- | --- | --- | --- |
| Chromatin Remodeling protein | 03875 | Yes | PP (RIP) | Adhvaryu et al. [[36](#_ENREF_36)] |
| Snf5 (Swi/Snf chromatin remodeling complex) | 00421 | Yes | PP | Fu et al. [[35](#_ENREF_35)] |
| GCN5 | 10847 | Yes | PP (RIP) | Adhvaryu et al. [[36](#_ENREF_36)] |
| Hp1 heterochromatin protein | 04017 | Yes | PP (RIP) | Freitag et al. [[130](#_ENREF_130)] |
| SAGA component | 04459 | Yes | PP (RIP) | Adhvaryu et al. [[36](#_ENREF_36)] |
| Histone H3 lysine methyl transferase-2 | 00269 | Yes | PP | Adhvaryu et al [[36](#_ENREF_36)] |
| SAGA component Sca7 | 07579 | Yes | Yes | This report |
| SDS3 histone deacetylase complex | 01599 | Yes | Yes | This report |
| Histone-like Transcription Factor | 06405 | Yes | -- | This report |
| Pbd-1 (BAH domain chromatin binding protein) | 02354 | Yes | -- | This report |
| Hir-1 (chromatin assembly protein) | 04035 | -- | -- |  |
| Set-1 histone methyl transferase | 01206 | -- | -- |  |
| Arid/bright domain containing protein – DNA binding protein | 05891 | Yes | -- |  |
| Histone acetyltransferase B subunit 2 | 06679 | -- | -- |  |
| Histone acetyltransferase | 00359 | -- | -- |  |
| Dap-1 protein | 04258 | -- | -- |  |
| SAGA component Spt3 | 07992 | Yes | -- |  |
| SPT8 transcription factor | 09208 | -- | -- |  |
