# Supplementary material for: Neurospora crassa Female Development Requires the PACC and Other Signal Transduction Pathways, Transcription Factors, Chromatin Remodeling, Cell-To-Cell Fusion, and Autophagy
Source: PLoS One. 2014 Oct 21;9(10):e110603. doi: 10.1371/journal.pone.0110603 (PMC4204872; doi:10.1371/journal.pone.0110603)
Supplement: Table S3 — Primers used in cloning PACC pathway genes. The primers used for PCR amplification and cloning of the PACC pathway genes are listed. The added restriction sites used for inserting the genes into pMB60 and pMB61 are underlined. The pacC-F, pacC-R, pacC-activated-F, and pacC-activated-R were used with the Gibson cloning kit to introduce a stop codon into the pacC gene. (DOCX) [file pone.0110603.s003.docx]

**Table S3. Primers used in cloning PACC pathway genes.**

| Primer name | Cloning into | Sequence (restriction sites underlined) |
| --- | --- | --- |
| palA-F | pBM61 | TAAACTAGTAGAGAGTGTAGCCAGAGTGAC |
| palA-R | pBM61 | TAATGGGCCCTACTCTTCAGTTGCTGACTC |
| palA-GFP-F | pMF272 | AATAGCGGCCGCAGAGAGTGTAGCCAGAGTGAC |
| palA-GFP-R | pMF272 | CATGTTAATTAACCCAAACCGGATCCCCTGAGAC |
| palA-GFP-ccg1-F | pMF272 | AATTAACTAGTCACCATGACAACCACCCACGTGCTG |
| palA-GFP-ccg1-R | pMF272 | CTCCTTAATTAACCCAAACCGGATCCCCTGAGAC |
| palB-F | pBM60 | TAAGGGCCCATGACGAGTAGCTGTACTAGC |
| palB-R | pBM60 | TAAGAATCCTTGCCACAGAGAAACCATCGC |
| palC-F | pBM61 | TAAACTAGTGACAAAGTGCCAGCAACGATC |
| palC-R | pBM61 | AATGAATTCATGAGGAGGAAGAGGAGCTTC |
| palF-F | pBM61 | ATCAGCGGCCGCGGAAAGGTTGTGATGGCATCC |
| palF-R | pBM61 | CAAATAGGATCCTTGCCACACATATGCAGAGCC |
| palH-F | pBM60 | ATATCCCGGGAAAGGAGAGGTGTTGCAGTGC |
| palH-R | pBM60 | TTAACTAGTTCCAAGACCATGCACTCTCTC |
| pacC-F | pBM60 | GAGGTCGACGGTATCGATAAGCTTGATATCGAATTC  GCAGATGCCATCTACAACC |
| pacC-R | pBM60 | GAATTGGAGCTCCACCGCGGTGGCGGCCGCTCTAGA  CAGCCTTCCATCGGTGGATGC |
| pacC-activated-F | pBM60 | AACGAGCGCCGCTAGGATCCCGGTGTCCTGCAAAGCGCG |
| pacC-activated-R | pBM60 | TTGCAGGACACCGGGATCCTAGCGGCGCTCGTTATGG |
